# Traditional Uses, Phytochemical Constituents and Pharmacological Properties of *Averrhoa carambola* L.: A Review

**DOI:** 10.3389/fphar.2021.699899

**Published:** 2021-08-12

**Authors:** Fei Luan, Lixia Peng, Ziqin Lei, Xiyu Jia, Junbo Zou, Yan Yang, Xirui He, Nan Zeng

**Affiliations:** ^1^ State Key Laboratory of Southwestern Chinese Medicine Resources, School of Pharmacy, Chengdu University of Traditional Chinese Medicine, Chengdu, China; ^2^ Department of Pharmacology, College of Pharmacy, Shaanxi University of Chinese Medicine, Xianyang, China; ^3^ Department of Bioengineering, Zhuhai Campus of Zunyi Medical University, Zhuhai, China

**Keywords:** *Averrhoa carambola*, chemical composition, antidiabetic, anticancer, toxicology

## Abstract

*Averrhoa carambola* L. (star fruit) is an edible fruit that is extensively cultivated in southern China, Southeast Asia, India, and northern South America. It has a sweet and juicy taste and is frequently used in fruit salads and fruit platters, as a garnish in cocktail drinks and beverages, or squeezed into juice and served as a beverage. Traditionally, it has been used for treating diabetes and diabetic nephropathy, arthralgia, vomiting, lithangiuria, coughing, hangovers, and chronic paroxysmal headache for thousands of years. Currently, approximately **132** compounds have been isolated from *A. carambola*. Among them, flavonoids, benzoquinone, and their glycosides have been considered as biologically active substances, which are responsible for various biological activities. Pharmacological studies have revealed that crude extracts or monomeric compounds from *A. carambola* exhibit multiple bioactivities, such as anti-oxidant, anti-hyperglycemic, anti-obesity, anti-hyperlipidemic, anti-tumor, anti-inflammatory, hepatoprotective, cardioprotective, anti-hypertensive, neuroprotective, and others. Thus, *A. carambola* is a valuable treatment in Chinese medicine with therapeutic potential for multiple diseases, especially diabetes and diabetes-related diseases. Even though it is a very promising candidate in the development of functional food and the pharmaceutical industry, reports on its bioactivities have only been conducted *in vivo* and *in vitro* and there is a gap in research regarding clinical settings and safety. This review therefore provides a comprehensive and systematic overview of current progress on botany, ethnopharmacology, phytochemistry, pharmacology, and toxicity of *A. carambola*, providing a valuable reference for further developments and applications of *A. carambola* in the pharmaceutical industry and functional food.

## Introduction

**FIGURE 1:**
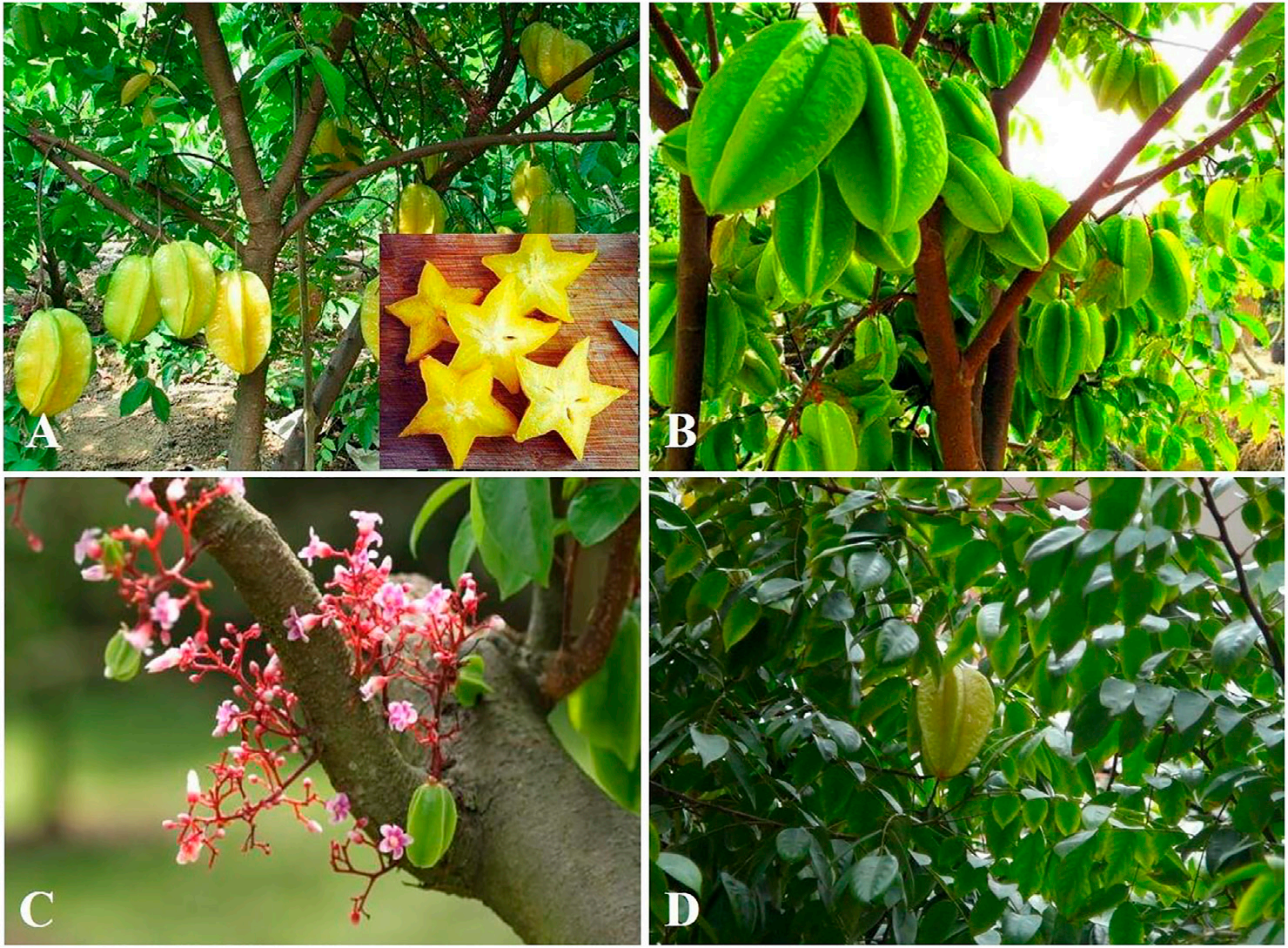
*A. carambola*: **(A)**: whole plants; **(B)**: fruits; **(C)**: flowers and woods; **(D)**: leaves (https://image.baidu.com/).

**FIGURE 2:**
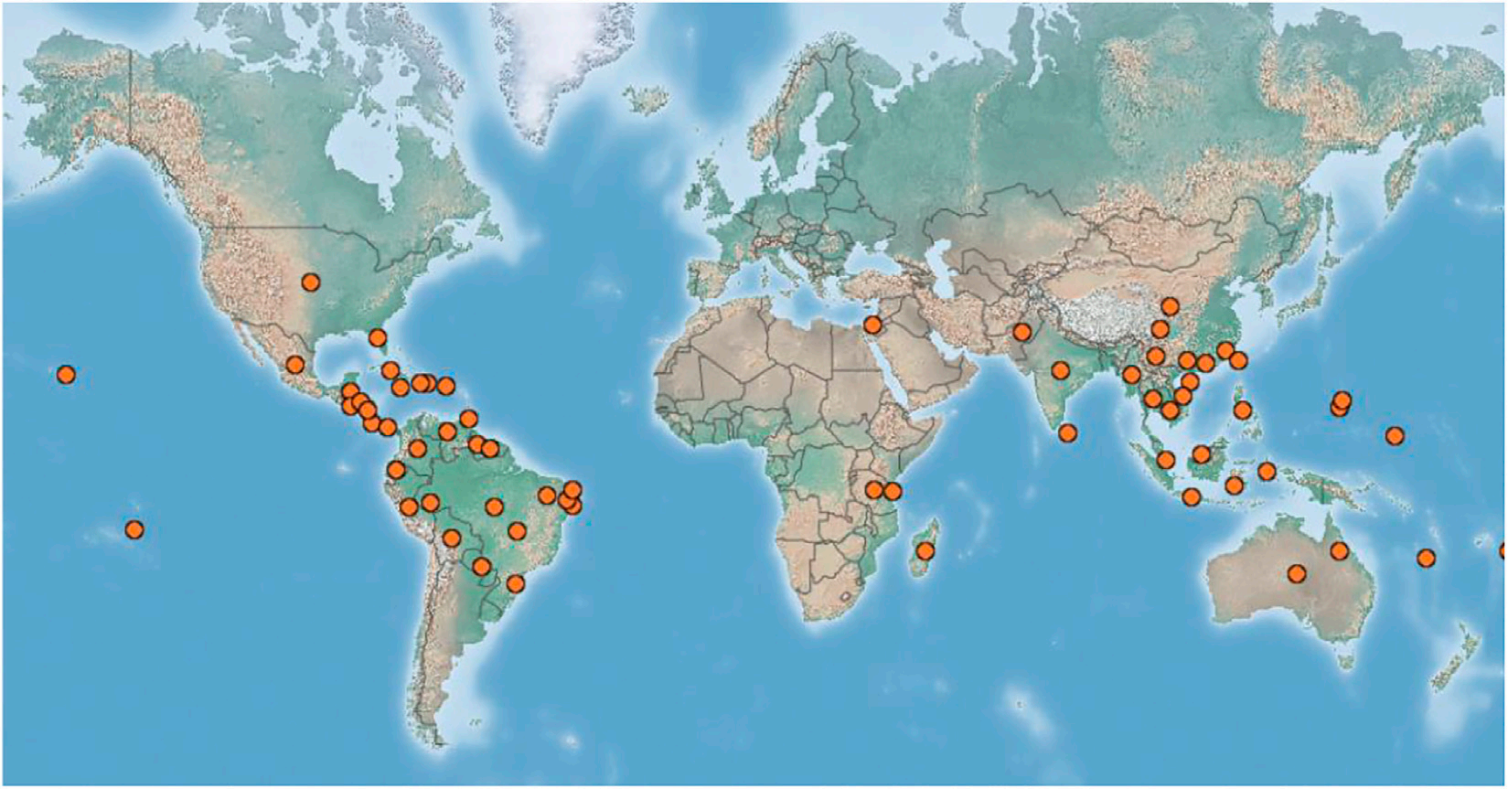
Geographical distribution of *A. carambola* throughout the world (https://www.cabi.org/isc/datasheet/8082).

*Averrhoa carambola* L., commonly known as carambola or star fruit, is a perennial tree in the family Oxalidaceae (Figure 1). It is considered native to Malaysia, however, it is a tropical American species introduced to Asia by the Spanish galleons and mainly cultivated throughout tropical and warm subtropical areas (Figure 2). The fruit is of high commercial value and is specially and extensively distributed and cultivated in southern China, Southeast Asia, India, and northern South America (Saikia et al., 2015; Leivas et al., 2016b; Varela-Martínez et al., 2019; Zulfajri et al., 2019). *A. carambola* is fleshy, crunchy, juicy, slightly tart, acidic, and sweet in the taste. It is star-shaped and golden-yellow in appearance and is frequently used in the preparation of fruit salads and fruit platters, as a garnish in cocktails and beverages, or squeezed into a juice and served as a functional beverage. It is also used in jellies, ice creams, preserves, and sweets owing to its high moisture content and highly perishability, especially in tropical regions such as Malaysia, Singapore, and Indonesia (Valim et al., 2016; Chua et al., 2017; Huynh and Nguyen, 2017; Jia et al., 2018; Lu et al., 2018). For instance, in Malaysia star fruits are usually blended with apples and braised with cloves and sugars or cooked along with meat or seafood (Bhat et al., 2011). Generally, star fruits are regarded as an abundant source of various nutrients such as minerals, proteins, and vitamins, and also rich in natural phytochemicals such as flavonoids, terpenes, saponins, alkaloids, proanthocyanidins, vitamin C, tartaric acid, oxalic acid, α-ketoglutaric acid, citric acid, vitamin B_1_ and B_2_, carotene, pectin, cellulose, gallic acid, epicatechin, fatty acids, volatile flavors, fibers, hemicellulose, polysaccharides, and sterols (Shui and Leong, 2006; Benkeblia and Lopez, 2015; Leivas et al., 2015; Muthu et al., 2016; Yang et al., 2017; Zulfajri et al., 2019). Simultaneously, GC-MS analysis has demonstrated that the abundant fatty acids existing in *A. carambola* leaves were α-linolenic acid (62.04%) and oleic acid (55.44%) in fruits. Moreover, the proportion of total unsaturated fatty acids existing both in the fruits and leaves of *A. carambola* comprise more than 77% of total fatty acid (Wei et al., 2014). The fructose content (38–48%) and glucose content (21–25%) have predominantly sugar-based compositions in *A. carambola* ripe fruits, while sorbitol is also another major sugar alcohol (2.4–10.5%) in ripe fruits (Ramadan et al., 2020). Additionally, the presence of high amounts of fibers in this plant contributes to the absorption of glucose, restraint glucose diffusion into the bloodstream, and maintain normal blood glucose levels (Fan et al., 2020). Furthermore, the byproduct or pomace residue from *A. carambola* left after juice drink extraction contains more antioxidants than the extracted juice (Shui and Leong, 2006). Interestingly, incorporation of 4% *A. carambola* fruit juice and 6% *Bambusa polymorpha* Munro (Poaceae family) shoot extract, significantly prolonged the shelf life of pork nuggets by at least 2 weeks (Thomas et al., 2016). Recent studies found that an antifreeze protein purified from the cold acclimated leaves of *Drimys angustifolia* Miers (Winteraceae family) and synergistic pectin-maltodextrin-sodium chloride edible coating could dramatically increase the quality of frozen *A. carambola* (Provesi et al., 2019; Mohd Suhaimi et al., 2021).

In recent years, phytochemical investigations have revealed that the major chemical components of *A. carambola* mainly include flavonoids, terpenes, and other phenolics. Among them, 2-dodecyl-6-methoxycyclohexa-2,5-diene-1,4-dione **(122, DMDD)** is the most representative chemical compound with multiple biological activities (Gao et al., 2015; Xie et al., 2016; Chen et al., 2017b). Pharmacological studies have demonstrated that the crude extracts or active substances of *A. carambola* have multiple health-promoting effects, and many of the biological effects above mentioned have ethnomedicinal uses. Furthermore, the usable range of *A. carambola* is increasingly expanding from medicinal plants to ornamental plants in gardens. For instance, *A. carambola* is widely planted as a decorative tree in the streets of southern Chinese cities because of its beautiful appearance (Wu et al., 2020b). More importantly, it is reported that the total consumption per year of *A. carambola* in China is about 2.6 million tons, whereas the annual production of *A. carambola* is only two million tons (Wu et al., 2020b).

To date, there has been no authoritative published systematic and comprehensive review that focuses on all of the important aspects of *A. carambola*. In the present review, recent advances in traditional uses, botanical characteristics, distribution, taxonomy, phytochemical constituents, biological effects as well as the toxicities of *A. carambola* are comprehensively presented and critically evaluated. Furthermore, the underlying mechanism associated with the bioactivities of crude extracts or components from this plant is also well summarized. The review is helpful for researchers by providing a comprehensive understanding of this increasingly important herb and provides a scientific basis for further study and exploitation of medicinal agents or functional food from *A. carambola* in the future.

## Material and Methods

This review collected, analyzed, summarized literature on the botanical description, traditional uses, chemical constituents, pharmacological activities, and toxicities of *A. carambola*. All information was systematically gathered from globally accepted scientific databases by Internet databases, including Elsevier, ScienceDirect, PubMed, Web of Science, Wiley, Springer, SciFinder, ACS Publications, CNKI, WanFang, Google Scholar, Baidu Scholar, The Plant List Database, and other literature sources (Ph.D. and MSc dissertations). All published contributions on *A. carambola* in different languages were included and cited. The identification and examination of the collected works were based on titles and abstracts. The reference lists of the retrieved publications were also checked to identify further relevant papers. The chemical structures of all isolated compounds were drawn by using ChemBioDraw Ultra 14.0 software.

## Botanical Description, Geographic Distribution, and Taxonomy

### Botanical Description

Botanically, *A. carambola* is a medium-sized tree reaching up to 3–15 m tall. The stem is gray bark. The leaf is odd-numbered compound leaves, alternate, leaflets 5-13, entire, ovoid, or elliptic, 3–7 cm long, acuminate at apex, with a round base, skewed on one side, sparsely pilose or glabrous underneath. The flower is cymes or panicles; sepals 5, about 5 mm long, arranged in imbricate shape, synthetic ring at the base; petals slightly dorsal, 0.8–1 cm long, purple-red, sometimes pink or white on the back; stamens 5-10; Ovary 5 compartments, many ovules in each compartment, style 5. The fruit is fleshy, drooping, with 5 edges, rarely 6 or 3 edges, star-shaped in cross section, 5–8 cm long, light green or waxy yellow, sometimes dark red. The seed is dark brown (Flora of China Editorial Committee, 1998).

### Geographic Distribution

*A. carambola* is traditionally considered to originate from Malaysia, although it has also been speculated to be a tropical American species introduced to Asia by the Spanish galleons. *A. carambola* has a wider climate range and can grow within the latitudinal range from 32°N to 30°S and withstand growing in both the hot humid tropics and subtropical countries including Egypt and Israel, and can tolerate short periods of freezing temperatures as low as −3°C. It prefers well-drained soils ideally between pH 5.5–6.5 but can tolerate pH between 5 and 8.5 (Bircher and Bircher, 2000). In recent years, it has been extensively distributed and widely cultivated in most parts of the world (Figure 2), e.g., Asia countries including China and India, Africa countries including Madagascar and Tanzania, North America countries including Mexico and Honduras, Oceania countries including Australia and French Polynesia, South America countries including Brazil and Bolivia, etc. (https://www.cabi.org/isc/datasheet/8082).

### Taxonomy

*A. carambola* belongs to the family Oxalidaceae, which consists of over 900 species belonging to seven genera, such as Dapania, Oxalis, Sarcotheca, Eichleria, Biophytum, Hypseocharis, and Averrhoa. Among them, the genus *Averrhoa* mainly includes three species, namely *A. carambola*, *A. bilimbi* L., and *A. dolichocarpa* Ruhayah and Sunart (https://www.cabi.org/isc/datasheet/8082; Moresco et al., 2012). Importantly, *A. carambola* is commonly known as star fruit or carambola, bearing deeply ridged, yellow-brown, edible fruit.

## Traditional Uses

*A. carambola* has been traditionally used for thousands of years in treating diabetes and diabetic nephropathy (DN), arthralgia, vomiting, lithangiuria, coughing and hangovers, and chronic paroxysmal headache. The different medicinal organs of *A. carambola* including leaves, roots, flowers, and the fruits have been utilized as ethnomedicine in Chinese, Indian, Malaysian, and Brazilian medicine for a long time. For instance, the crushed shoots or leaves of *A. carambola* are commonly applied in traditional Malaysia medicine to treat headache, chicken-pox, and ringworm, while a decoction of the leaves and fruits of *A. carambola* is generally used for treating vomiting, fevers, aphthous stomatitis, and angina (Yang et al., 2020b). In Sri Lanka, *A. carambola* fruits are traditionally used to treat and prevent diabetes mellitus due to their excellent hypoglycemic effects (Abeysekera et al., 2015). In traditional Brazilian medicine, the fruit, juice, as well as tea made from leaves of *A. carambola* have been traditionally utilized to prevent and treat diabetes, high blood pressure, and urinary system diseases and *A. carambola* is also considered as a food supplement that can improve the appetite of people with poor appetite (Vasconcelos et al., 2006; Soncini et al., 2011). The traditional Indian medicine records that the ripe fruits of *A. carambola* can be used for effectively curing the hemorrhage of hemorrhoids and it is also regarded as a remedy for the treatment of eczema, fever, and diarrhea. Furthermore, the ripe fruit of *A. carambola* is mainly considered as digestive and tonic in Ayurveda (Vasant and Narasimhacharya, 2014). As a Traditional Chinese Medicine (TCM), the roots, fruits, and leaves of *A. carambola* have been increasingly recognized as an effective herbal medicine in invigorating kidney function and reinforcing *Yang* (it refers to the masculine, active and positive principle) and is used for the treatment of various ailments with a long history (World Health Organization and Regional Office for the Western Pacific, 2007; Wei et al., 2018). More specifically, the roots of *A. carambola* have been commonly accepted as a diuretic and appetite stimulant agent, it is also used as an antidiarrheal and febrifugal drug with a long history of medical use in TCM for the treatment of arthralgia, diabetes, DN, lithangiuria, and chronic paroxysmal headache in ancient times (Cabrini et al., 2011; Wen et al., 2013; Zheng et al., 2013; Chen et al., 2017b). At the same time, *A. carambola* leaves have been commonly utilized for alleviating vomiting, headaches, diabetes, coughing, and hangovers for a many years (Carolino et al., 2005; Ferreira et al., 2008). Furthermore, *A. carambola* fruits are frequently applied to effectively remedy malarial splenomegaly and food poisoning caused by meat sources (Pang et al., 2017). Overall, the leaves, roots, flowers, and fruits of this plant, might be used as a dietary supplement and should be further studied and developed as a functional food or therapeutic agent in the management of human health.

## Nutritional and Phytochemical Composition

### Nutritional Composition

Nutrient substances, such as minerals, vitamins, cellulose, hemicelluloses, pectin, and others are contained in the fruit of *A. carambola*. It has been reported that *A. carambola* contains cellulose (60%), hemicelluloses (27%), and pectin (13%), which may contribute to controlling blood sugar levels (Lakmal et al., 2021). Moreover, carotene, vitamins, and acids were found in the ripe fruit of *A. carambola*, with high levels of vitamin C (25.8 mg/100 g fruit), tartaric acid (4.37 mg/100 g fruit), vitamin B1 and B2 (0.12 mg/100 g fruit) (Muthu et al., 2016). Furthermore, mineral elements were also found to be contained in *A. carambola*, with high levels of potassium (167.13–168.0 mg/100 g fruit), phosphorous (17.87–17.88 mg/100 g fruit), magnesium (11.85–12.05 mg/100 g fruit), calcium (6.37–6.40 mg/100 g fruit), sodium (3.8–3.85 mg/100 g fruit), iron (0.34–0.45 mg/100 g fruit), zinc (0.29–0.51 mg/100 g fruit), copper (0.19–0.45 mg/100 g fruit), and manganese (0.04–0.52 mg/100 g fruit) (Muthu et al., 2016). These results indicate that *A. carambola* is low-calorie and may also have health-promoting properties.

### Phytochemical Compounds

**FIGURE 3:**
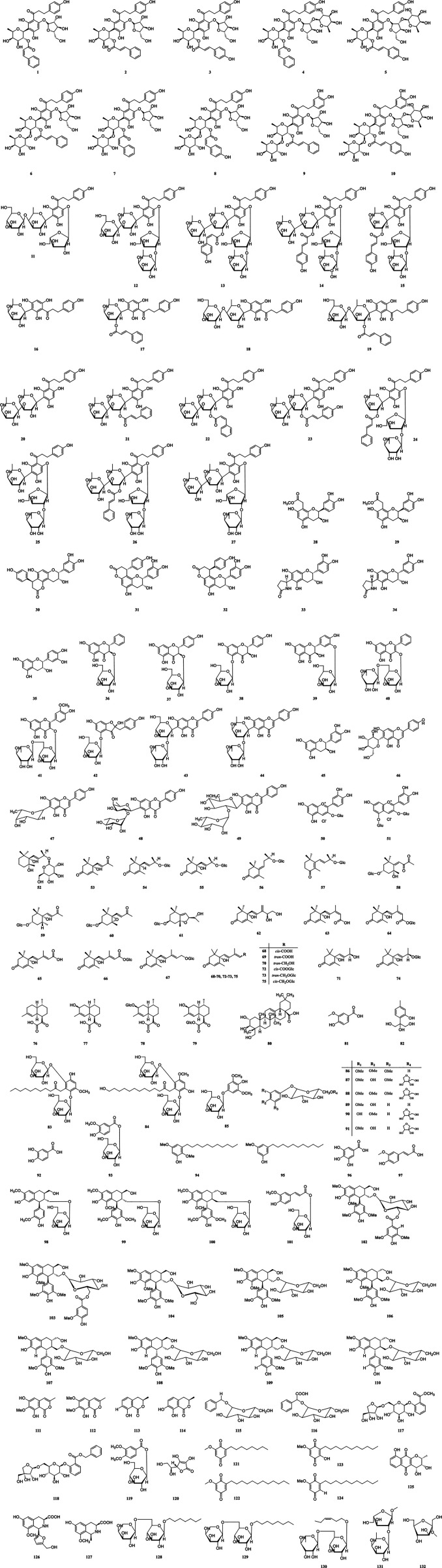
Chemical structures of compounds isolated from *A. carambola*.

Currently, approximately **132** phytochemical compounds have been separated and identified from *A. carambola*, which contains flavonoids, terpenes, phenylpropanoids, and their glycosides, among others. These include flavonoids, benzoquinone and their glycosides, which have been considered as the biologically active components responsible for multiple bioactivities. The compounds isolated from *A. carambola* are documented and listed in Table 1 and the chemical structures are drawn and presented in Figure 3.

**TABLE 1:** Chemical components isolated and structurally identified from *A. carambola*.

No.	Chemical constituents	Molecular formula	Extracts	Parts	References
**Flavonoids**					
1	Carambolaside R1	C_35_H_38_O_14_	EtOH	Leaves	Yang et al. (2020a)
2	Carambolaside R2	C_35_H_38_O_14_	EtOH	Leaves	Yang et al. (2020a)
3	Carambolaside R3	C_35_H_38_O_15_	EtOH	Leaves	Yang et al. (2020a)
4	Carambolaside S1	C_41_H_48_O_18_	EtOH	Leaves	Yang et al. (2020a)
5	Carambolaside S2	C_41_H_48_O_18_	EtOH	Leaves	Yang et al. (2020a)
6	Carambolaside T1	C_41_H_48_O_18_	EtOH	Leaves	Yang et al. (2020a)
7	Carambolaside T2	C_41_H_48_O_18_	EtOH	Leaves	Yang et al. (2020a)
8	Carambolaside T3	C_41_H_48_O_19_	EtOH	Leaves	Yang et al. (2020a)
9	3-Hydroxycarambolaside T1	C_41_H_48_O_19_	EtOH	Leaves	Yang et al. (2020a)
10	3-Hydroxycarambolaside P	C_47_H_58_O_24_	EtOH	Leaves	Yang et al. (2020a)
11	Carambolaside M	C_32_H_42_O_18_	EtOH	Fruits	Jia et al. (2018)
12	Carambolaside N	C_38_H_52_O_22_	EtOH	Fruits	Jia et al. (2018)
13	Carambolaside O	C_47_H_58_O_23_	EtOH	Fruits	Jia et al. (2018)
14	Carambolaside P	C_47_H_58_O_23_	EtOH	Fruits	Jia et al. (2018)
15	Carambolaside Q	C_41_H_48_O_19_	EtOH	Fruits	Jia et al. (2018)
16	Carambolaside A	C_21_H_24_O_9_	MeOH	Fruits	Yang et al. (2015)
17	Carambolaside B	C_30_H_30_O_10_	MeOH	Fruits	Yang et al. (2015)
18	Carambolaside C	C_27_H_34_O_14_	MeOH	Fruits	Yang et al. (2015)
19	Carambolaside D	C_36_H_40_O_15_	MeOH	Fruits	Yang et al. (2015)
20	Carambolaside E	C_27_H_34_O_13_	MeOH	Fruits	Yang et al. (2016)
21	Carambolaside F	C_36_H_40_O_14_	MeOH	Fruits	Yang et al. (2016)
22	Carambolaside G	C_36_H_40_O_14_	MeOH	Fruits	Yang et al. (2016)
23	Carambolaside H	C_36_H_40_O_15_	MeOH	Fruits	Yang et al. (2016)
24	Carambolaside I	C_41_H_48_O_18_	MeOH	Fruits	Yang et al. (2016)
25	Carambolaside Ia	C_41_H_48_O_18_	MeOH	Fruits	Yang et al. (2016)
26	Carambolaside J	C_47_H_58_O_22_	MeOH	Fruits	Yang et al. (2016)
27	Carambolaside Ja	C_47_H_58_O_22_	MeOH	Fruits	Yang et al. (2016)
28	8-carboxymethyl-(+)-epicatechin methyl ester	C_18_H_18_O_8_	EtOH	Fruits	Jia et al. (2018)
29	(+)-Epicatechin	C_15_H_14_O_6_	EtOH	Fruits	Jia et al. (2018)
30	Epicatechin-(5,6-bc)-4β-(p-hydroxyphenyl)-dihydro-2(3H)-pyranone	C_24_H_20_O_8_	EtOH	Leaves	Yang et al. (2020b)
31	Epicatechin-(7,8-bc)-4α-(p-hydroxyphenyl)-dihydro-2(3H)-pyranone	C_24_H_20_O_8_	EtOH	Leaves	Yang et al. (2020b)
32	Epicatechin-(7,8-bc)-4β-(p-hydroxyphenyl)-dihydro-2(3H)-pyranone	C_24_H_20_O_8_	EtOH	Leaves	Yang et al. (2020b)
33	6-(S-2-Pyrrolidinone-5-yl)-epicatechin	C_19_H_19_NO_7_	EtOH	Leaves	Yang et al. (2020b)
34	6-(R-2-pyrrolidinone-5-yl)-epicatechin	C_19_H_19_NO_7_	EtOH	Leaves	Yang et al. (2020b)
35	(–)-Epicatechin	C_15_H_14_O_6_	EtOAc	Fruits	Gunawardena et al. (2015)
36	Pinobanksin 3-O-β-D-glucoside	C_21_H_22_O_10_	EtOH	Fruits	Jia et al. (2018)
37	Aromadendrin 3-O-β-D-glucoside	C_21_H_22_O_11_	EtOH	Fruits	Jia et al. (2018)
38	Helicioside A	C_21_H_22_O_12_	EtOH	Fruits	Jia et al. (2018)
39	Taxifolin 3′-O-β-D-glucoside	C_21_H_22_O_12_	EtOH	Fruits	Jia et al. (2018)
40	Norathyriol	C_13_H_8_O_6_	EtOH	Fruits	Jia et al. (2018)
41	Isorhamnetin 3-O-rutinoside	C_28_H_32_O_16_	EtOH	Fruits	Jia et al. (2018)
42	Hovertichoside C	C_36_H_46_O_14_	MeOH	Fruits	Yang et al. (2015)
43	Isovitexin 2″-O-α-L-rhamnopyranoside	C_27_H_30_O_14_	MeOH	Fruits	Yang et al. (2015)
44	Carambolaflavone	C_27_H_30_O_13_	MeOH	Fruits	Yang et al. (2015)
45	(+)-Catechin	C_15_H_14_O_6_	Aqueous	Roots	Liao et al. (2019a)
46	Isovitexin	C_21_H_22_O_10_	EtOH	Leaves	Araho et al. (2005)
47	Carambolaflavone A	C_27_H_30_O_13_	EtOH	Leaves	Araho et al. (2005), Moresco et al. (2012), Wang et al. (2018)
48	Carambolaflavone B	C_27_H_30_O_13_	EtOH	Leaves	Araho et al. (2005), Moresco et al. (2012), Wang et al. (2018)
49	Apigenin 6-C-(2′′-O-α-L-rhamnopyranosyl)-β-D-glucopyranoside	C_36_H_37_O_18_	EtOH	Leaves	Moresco et al. (2012)
50	Cyanidin-3-O-β-D-glucoside	C_21_H_21_O_11_Cl	EtOH	Leaves	Gunasegaran (1992)
51	Cyanidin-3, 5-O-β-D-diglucoside	C_27_H_37_O_16_Cl	EtOH	Leaves	Gunasegaran (1992)
**Terpenes**					
52	(5R,6S,7E,9R)-5,6,9-trihydroxy-7-megastigmene 9-O-β-D-glucoside	C_19_H_34_O_8_	EtOH	Fruits	Jia et al. (2019)
53	Drovomifoliol	C_13_H_19_O_3_	EtOH	Fruits	Jia et al. (2019)
54	3-oxo-α-ionol-9-O-β-D-glucoside	C_19_H_31_O_7_	EtOH	Fruits	Jia et al. (2019)
55	Roseoside	C_19_H_30_O_8_	EtOH	Fruits	Jia et al. (2019)
56	3-oxo-9-O-β-D-glucosyloxy-4,6*E*-megastigmadien	C_19_H_30_O_8_	EtOH	Fruits	Jia et al. (2019)
57	4-oxo-β-ionol 9-O-β-D-glucoside	C_19_H_31_O_8_	EtOH	Fruits	Jia et al. (2019)
58	Cannabiside D	C_19_H_30_O_9_	EtOH	Fruits	Jia et al. (2019)
59	Dendranthemoside B	C_19_H_32_O_8_	EtOH	Fruits	Jia et al. (2019)
60	Icariside	C_27_H_30_O_11_	EtOH	Fruits	Jia et al. (2019)
61	Officinoside A	C_19_H_32_O_8_	EtOH	Fruits	Jia et al. (2019)
62	6*S*,7*E*,10*S*)-△^9,15^-10-hydroxyabscisic alcohol	C_15_H_22_O_4_	EtOH	Fruits	Jia et al. (2019)
63	Abscisic acid	C_15_H_20_O_4_	EtOH	Fruits	Jia et al. (2019)
64	Abscisyl β-D-glucoside	C_21_H_31_O_10_	EtOH	Fruits	Jia et al. (2019)
65	9*E*-abscisic acid	C_15_H_20_O_4_	EtOH	Fruits	Jia et al. (2019)
66	9*E*-abscisyl β-D-glucoside	C_21_H_32_O_10_	EtOH	Fruits	Jia et al. (2019)
67	9*E*-abscisic alcohol β-D-glucoside	C_21_H_33_O_9_	EtOH	Fruits	Jia et al. (2019)
68	*cis*-abscisic acid	C_15_H_20_O_4_	EtOAc	Fruits	Gunawardena et al. (2015)
69	*trans*-abscisic acid	C_15_H_20_O_4_	EtOAc	Fruits	Gunawardena et al. (2015)
70	*trans*-abscisic alcohol	C_15_H_22_O_3_	EtOAc	Fruits	Gunawardena et al. (2015)
71	(6*S*,9*R*)-vomifoliol	C_13_H_20_O_3_	EtOAc	Fruits	Gunawardena et al. (2015)
72	*cis*-abscisic acid β-D-glucopyranosyl ester	C_22_H_32_O_8_	EtOAc	Fruits	Gunawardena et al. (2015)
73	*trans*-abscisic alcohol β-D-glucopyranoside	C_22_H_33_O_7_	EtOAc	Fruits	Gunawardena et al. (2015)
74	(6*S*,9*R*)-roseoside	C_14_H_22_O_3_	EtOAc	Fruits	Gunawardena et al. (2015)
75	*cis*-abscisic alcohol β-D-glucopyranoside	C_22_H_33_O_7_	EtOAc	Fruits	Gunawardena et al. (2015)
76	Artemisinic acid	C_15_H_22_O_2_	EtOH	Fruits	Yang et al. (2012)
77	3-β-hydroxyartemisinic acid	C_15_H_22_O_3_	EtOH	Fruits	Yang et al. (2012)
78	Artemisinic acid 3-β-O-β-D-glucopyranoside	C_21_H_32_O_8_	EtOH	Fruits	Yang et al. (2012)
79	3-β-hydroxyartemisinic acid β-D-glucopyranosyl ester	C_21_H_32_O_8_	EtOH	Fruits	Yang et al. (2012)
80	Arjunolic acid	C_30_H_48_O_5_	MeOH	Fruits	Yang et al. (2014)
**Phenolics**					
81	Vanillic acid	C_8_H_8_O_4_	MeOH	Fruits	Yang et al. (2014)
82	8,9,10-trihydroxythymol	C_10_H_10_O_4_	MeOH	Fruits	Yang et al. (2014)
83	Carambolaside K	C_30_H_48_O_15_	EtOH	Fruits	Jia et al. (2017)
84	Carambolaside L	C_32_H_52_O_16_	EtOH	Fruits	Jia et al. (2017)
85	Koaburaside	C_14_H_20_O_9_	EtOH	Fruits	Jia et al. (2017)
86	3,4,5-trimethoxyphenol-1-O-β-D-glucopyranoside	C_15_H_22_O_9_	BuOH	Roots	Wen et al. (2012)
87	3,5-dimethoxy-4-hydroxyphenyl 1-O-β-apiofuranosyl (1''→6')-O-β-D-glucopyranoside	C_19_H_28_O_13_	BuOH	Roots	Wen et al. (2012)
88	3,4,5-trimethoxyphenyl 1-O-β-apiofuranosyl (1''→6')-β-glucopyranoside	C_20_H_30_O_13_	BuOH	Roots	Wen et al. (2012)
89	Methoxyhydroquinone-4-β-D-glucopyranoside	C_13_H_18_O_18_	BuOH	Roots	Wen et al. (2012)
90	3-hydroxy-4-methoxyphenol 1-O-β-D-apiofuranosyl-(1''→6')-O-β-D-glucopyranoside	C_18_H_26_O_12_	BuOH	Roots	Wen et al. (2012)
91	4-hydroxy-3-methoxyphenol 1-O-β-D-apiofuranosyl-(1''→6')-O-β-D-glucopyranoside	C_18_H_26_O_12_	BuOH	Roots	Wen et al. (2012)
92	Protocatechuic acid	C_7_H_6_O_4_	EtOH	Fruits	Jia et al. (2017)
93	1-*O*-vanilloyl-β-D-glucose	C_14_H_18_O_9_	EtOH	Fruits	Jia et al. (2017)
94	2,5-dimethoxy-3-undecylphenol	C_19_H_33_O_3_	EtOAc	Wood	Chakthong et al. (2010)
95	5-methoxy-3-undecylphenol	C_18_H_31_O_2_	EtOAc	Wood	Chakthong et al. (2010)
96	Gallic acid	C_7_H_6_O_5_	Acetone	Fruits	Shui and Leong (2004)
**Phenylpropanoids**					
97	Ferulic acid	C_10_H_10_O_4_	MeOH	Fruits	Yang et al. (2014)
98	(+)-isolariciresinol 9-O-β-D-glucoside	C_27_H_36_O_12_	EtOH	Fruits	Jia et al. (2017)
99	(+)-lyoniresinol 9-O-β-D-glucoside	C_28_H_38_O_13_	EtOH	Fruits	Jia et al. (2017)
100	(−)-lyoniresinol 9-O-β-D-glucoside	C_28_H_38_O_13_	EtOH	Fruits	Jia et al. (2017)
101	1-O-feruloyl-β-D-glucose	C_16_H_20_O_9_	EtOH	Fruits	Jia et al. (2017)
102	Tarennanosides A	C_37_H_46_O_17_	Aqueous	Roots	Liao et al. (2019a)
103	Fernandoside	C_36_H_44_O_16_	Aqueous	Roots	Liao et al. (2019a)
104	7α-[(β-glucopyranosyl) oxy]-lyoniresinol	C_28_H_38_O_13_	Aqueous	Roots	Liao et al. (2019a)
105	(+)-lyoniresinol 3α-O-β-D-glucopyranoside	C_28_H_38_O_13_	Aqueous	Roots	Liao et al. (2019a)
106	(−)-lyoniresinol 3α-O-β-D-glucopyranoside	C_28_H_38_O_13_	Aqueous	Roots	Liao et al. (2019a)
107	(−)-5′-methoxy-isolariciresinol 3α-O-β-D-glucopyranoside	C_27_H_36_O_12_	Aqueous	Roots	Liao et al. (2019a)
108	(+)-5′-methoxy-isolariciresinol 3α-O-β-D-glucopyranoside	C_27_H_36_O_12_	BuOH	Roots	Wen et al. (2012)
109	(+)-isolariciresinol 3α-O-β-D-glucopyranoside	C_26_H_34_O_11_	BuOH	Roots	Wen et al. (2012)
110	(−)-isolariciresinol 3α-O-β-D-glucopyranoside	C_26_H_34_O_11_	BuOH	Roots	Wen et al. (2012)
111	Reticulol	C_11_H_10_O_5_	EtOAc	Fruits	Sritharan et al. (2019)
112	6-*O*-methyl-reticulol	C_11_H_10_O_5_	EtOAc	Fruits	Sritharan et al. (2019)
113	5-methylmellein	C_11_H_12_O_3_	EtOAc	Fruits	Sritharan et al. (2019)
114	7-hydroxy-5-methylmellein	C_11_H_12_O_4_	EtOAc	Fruits	Sritharan et al. (2019)
**Other constituents**					
115	Benzyl-1-O-β-D-glucopyranoside	C_13_H_18_O_6_	BuOH	Roots	Wen et al. (2012)
116	(2S)-2-O-β-D-glucopyranosyl-2-hydroxyphenyl-acetic acid	C_14_H_18_O_8_	BuOH	Roots	Wen et al. (2012)
117	Methyl 2-β-D-apiofuranosyl-(1→6)-β-D-glucopyranosyloxybenzoate	C_19_H_26_O_12_	EtOH	Leaves	Yang et al. (2020b)
118	Benzyl 2-β-D-apiofuranosyl-(1→6)-β-D-glucopyranosyloxybenzoate	C_25_H_30_O_12_	EtOH	Leaves	Yang et al. (2020b)
119	Tecomin	C_15_H_20_O_9_	EtOH	Fruits	Jia et al. (2017)
120	L-ascorbic acid	C_6_H_8_O_6_	Acetone	Fruits	Shui and Leong (2004)
121	2-methoxy-6-nonyl-cyclohexa-2,5-diene-1,4-dione	C_16_H_24_O_3_	EtOH	Roots	Wen et al. (2014)
122	2-dodecyl-6-methoxycyclohexa-2,5-diene-1,4-dione	C_19_H_30_O_3_	EtOH	Roots	Zhang et al. (2020)
123	5-*O*-methylembelin	C_18_H_28_O_4_	EtOAc	Woods	Chakthong et al. (2010)
124	2-dehydroxy-5-*O*-methylembelin	C_18_H_28_O_3_	EtOAc	Woods	Chakthong et al. (2010)
125	(+)-cryptosporin	C_14_H_12_O_6_	EtOH	Fruits	Jia et al. (2017)
126	(1R*,3S*)-1-(5-hydroxymethylfuran-2-yl)-3-carboxy-6-hydroxy-8-methoxyl-1,2,3,4-tetrahydroisoquinoline	C_16_H_17_NO_6_	MeOH	Fruits	Yang et al. (2014)
127	(1S*,3S*)-1-methyl-3-carboxy-6-hydroxy-8-methyoxyl-1,2,3,4-tetrahydroisoquinoline	C_12_H_15_NO_4_	MeOH	Fruits	Yang et al. (2014)
128	Heptyl vicianoside	C_18_H_34_O_10_	EtOH	Fruits	Yang et al. (2019b)
129	Octyl vicianoside	C_21_H_30_O_11_	EtOH	Fruits	Yang et al. (2019b)
130	*cis*-3-hexenyl rutinoside	C_34_H_43_O_16_	EtOH	Fruits	Yang et al. (2019b)
131	Methyl 2-*O*-β-D-fucopyranosyl-α-L-arabinofuranoside	C_12_H_22_O_9_	EtOH	Fruits	Yang et al. (2019b)
132	Methyl α-D-fructofuranoside	C_7_H_14_O_6_	EtOH	Fruits	Yang et al. (2019b)

EtOH, ethanol; EtOAc, ethyl acetate; *n*-BuOH, *n*-butanol.

#### Flavonoids

Various studies have found that the flavonoids isolated from this plant possess excellent antioxidant and radical scavenging properties, which can be used to prevent and treat the occurrence of chronic and cardiovascular illness (Maqsood et al., 2020). Until now, **51** flavonoids **(1–51)** have been separated and characterized by nuclear magnetic resonance (NMR) and mass spectrometer (MS) technologies from the leaves, fruits, and roots of *A. carambola*. Among these are compounds **(1–27)** that are dihydrochalcone C-glycosides and other compounds **(28–35)** are flavan-3-ols, of which all exhibited significant radical scavenging activities against the DPPH and ABTS, while some compounds **(36–51)** are other types with multiple structures. Both the compounds carambolaflavone A **(47)** and carambolaflavone B **(48)** showed excellent antihyperglycemic activity both in hyperglycemic and diabetic rats’ model (Cazarolli et al., 2009; Cazarolli et al., 2012). Afterward, Wang et al. (2018) conducted the total synthesis of the enantiomers of carambolaflavone A **(47)** and found that structurally the β-fucosyl moiety absolute configuration was D instead of L. Information of these isolated flavonoids is listed in Table 1. Their chemical structures were drawn by ChemBioDraw Ultra 14.0 and are described in Figure 3.

#### Terpenes

Terpenes are a group of secondary metabolites in plants that consist of one or more isoprene subunits (Bahramsoltani et al., 2020). They have the function of promoting the coloring in many and various vegetables and fruits (Farias et al., 2020). To date, **29** terpenes **(52–80)** have been mainly separated and identified from the fresh fruits of *A. carambola*. Gunawardena et al. (2015) analyzed different terpenes in star fruits using NMR and MS methods, and the major terpenes identified were *cis*-abscisic acid (**68**, 12 mg), *trans*-abscisic acid (**69**, 3.5 mg), *trans*-abscisic alcohol (**70**, 12 mg), (6S,9R)-vomifoliol (**71**, 8.5 mg), *cis*-abscisic acid β-D-glucopyranosyl ester (**72**, 19 mg), *trans*-abscisic alcohol β-D-glucopyranoside (**73**, 12 mg), (6S,9R)-roseoside (**74**, 12 mg), and *cis*-abscisic alcohol β-D-glucopyranoside (**75**, 113 mg). Moreover, Jia et al. (2019) found that the terpenes-derived components from star fruits are primarily C_13_- and C_15_-norisoprenoids, which tremendously strengthen the flavor of *A. carambola* fruits. Information of these terpenes is listed in Table 1. The chemical structures were draw by ChemBioDraw Ultra 14.0 and are shown in Figure 3. However, the pharmacological activities of most terpenes is still unclear.

#### Phenolics

Phenolic compositions are represented as one of the major classes of plant secondary metabolites and extensively dispersed among plant parts, phytochemical studies have found that these compounds principally exist in the roots and fruits of *A. carambola* (Wen et al., 2012; Yang et al., 2014; Jia et al., 2017; Liao et al., 2019a). At present, **16** phenolic components **(81–96)** were isolated and characterized by FT-IR, ^1^H-NMR, and ^13^C-NMR, from the roots and fruits of *A. carambola* with excellent antioxidant properties. Among them, compounds **83**, **84**, **94,** and **95** are alkyl phenols in structure. Pang et al. (2016) compared the phenolic and flavonoid compound content of four popular cultivars of *A. carambola* that originated from southern China and found that the contents of bound, free, and total phenolic for four cultivars were 6.4–19.7, 162.5–286.8, and 174.5–293.1 mg gallic acid equivalents per 100 g fresh weight, respectively. The contents of bound, free, and total flavonoid of the four cultivars were 1.1–7.8, 100.7–234.0, and 104.4–235.1 mg catechin equivalents per 100 g fresh weight, respectively, which indicated that a certain amount of non-flavonoid phenolic substances exist. These phenolic compositions are summarized in Table. Their chemical structures were drawn by ChemBioDraw Ultra 14.0 and are presented in Figure 3.

#### Phenylpropanoids

Phenylpropanoids are a kind of plant-derived organic compound, and these compounds are mainly derived from phenylalanine and tyrosine (Yao et al., 2020). At present, **18** phenylpropanoids **(97–114)** have been successfully separated and chemically identified by analyses of spectroscopic data including ^1^H-NMR and ^13^C-NMR, from the fruits and roots of *A. carambola*. These compounds can be classified as simple phenylpropanoids, lignans, and coumarins based on their substructure type. Among them, four simple phenylpropanoids **(97–101)** have been reported for the *A. carambola* fruit, twelve lignans **(102–110)** were primarily achieved and identified from *A. carambola* roots. Furthermore, four coumarins **(111–114)** have been found in *A. carambola* fruit, compounds reticulol **(111)** and 6-O-methyl-reticulol **(112)** are isocoumarins in structure, 5-methylmellein **(113)** and 7-hydroxy-5- methylmellein **(114)** are dihydroisocoumarins in structure. Reticulol **(111)** displayed moderate antioxidant capacity against DPPH with the IC_50_ value of 58 μg/ml (Sritharan et al., 2019). These phenylpropanoid constituents are summarized in Table 1 and the corresponding chemical structures were also draw by ChemBioDraw Ultra 14.0 and are presented in Figure 3.

#### Other Compounds

Up to date, apart from the chemical compounds listed above, few compounds **(115–132)** have been investigated and summarized in Table 1 and the corresponding chemical structures were presented in Figure 3. Briefly, compounds **117** and **118** are identified as benzoic acid, compounds **121–125** are quinones, compounds **126** and **127** are tetrahydroisoquinoline alkaloids, and components **128–132** are identified as alkyl glycosides. Among them, 2-dodecyl-6-methoxycyclohexa-2,5-diene-1,4-dione **(122)** was obtained and characterized from *A. carambola* roots showing multiple bioactive properties both in cell and animal experimental studies, including anti-cancer (Gao et al., 2015; Chen et al., 2017b), anti-diabetic nephropathy (Lu et al., 2019), anti-obesity (Li et al., 2016), anti-hyperglycemic (Zhang et al., 2020), anti-inflammatory (Xie et al., 2016), and neuroprotective activities (Wei et al., 2018).

## Pharmacological Activities

### Antioxidant Activity

Among pharmacologically active substances, natural antioxidants have gained widespread attention because they are safe and have low toxicity, and promising biological functions (Rufino et al., 2011). *A. carambola* leaves (ACL) supposedly exhibit the most potent antioxidant activities determined by DPPH, FRAP, and TEAC assays (Table 2). Phytochemical studies have shown that these leaves are rich in phenolic and flavonoid components, which are closely associated with antioxidant effects; this indicates that ACL is potentially an abundant source of natural antioxidants and could help prevent and treat oxidative stress-related diseases (Chen et al., 2017a). In a systematic comparison between twenty locally available fruits planted in Sri Lanka, Silva and Sirasa (2018) demonstrated that *A. carambola* had the third most potent antioxidant properties based on assays against FRAP and DPPH activities, total flavonoid content, total phenolic content, and vitamin C content. Siddika et al. (2020) found that the methanol extract of *A. carambola* leaves (MEACL, at 50–375 μg/ml) demonstrated dose-dependent moderate antioxidant activity when assayed against DPPH and ABTS^+^, with IC_50_ values of 62.0 and 6.0 μg/ml, respectively. Other phytochemical investigations have shown MEACL is rich in phenolics, which could be the drive behind its radical scavenging activity. Using paper spray ionization (PSI) coupled to high-resolution mass spectrometry, one study revealed that a bioactive compound, norathyriol, isolated from ethanol extracts of the bark of *A. carambola* (EEBAC) had antioxidant properties. EEBAC (at concentrations of 1.0, 3.0, 10, 30, and 100 μg/ml) displayed concentration-dependent antioxidant characteristics *via* suppressing the activities of α-glucosidase, elastase, ABTS^+^, DPPH, and tyrosinase enzyme, with IC_50_ values of 7.15, 20.34, 26.29, 55.55, and 56.46 μg/ml, respectively (Islam et al., 2020). Compound (**40**) at 2.5, 5, and 10 μg/ml, has shown powerful antioxidant effects against DPPH and ABTS^+^, with corresponding IC_50_ values of 4.9 and 9.63 μg/ml (Islam et al., 2020). The efficiency and potency of crude extracts or bioactive ingredients from *A. carambola* suggest it is a promising antioxidant in the pharmaceutical and functional food industries.

**TABLE 2:** Pharmacological effects of crude extracts and bioactive compounds of *A. carambola*.

Pharmacological activity	Compounds/Extracts	Types	Testing subjects	Doses/Duration	Effects/Mechanisms	References
**Antioxidant activity**						
	MEACL	*In vitro*	DPPH and ABTS^+^ assays	50–375 μg/ml	Showed moderate free radical scavenging activity against DPPH and ABTS^+^ free radical with the IC_50_ were 62.0 and 6.0 μg/ml, respectively	Siddika et al. (2020)
	PRAC	*In vitro*	DPPH and ABTS^+^ assays	0.5–2.5 mg/ml	Showed significant free radical scavenging activity against DPPH and ABTS^+^ with the IC_50_ of 0.10 and 0.33 mg/ml, respectively	Liao et al. (2019b)
	EEBAC	*In vitro*	α-glucosidase, elastase, ABTS+, DPPH, and tyrosinase enzyme assays	1.0, 3.0, 10, 30, and 100 μg/ml	Showed significant antioxidant activity, and the IC_50_ values were 7.15, 20.34, 26.29, 55.55 and 56.46 μg/ml, respectively	Islam et al. (2020)
	40	*In vitro*	DPPH and ABTS^+^ assays	2.5, 5, and 10 μg/ml	Showed significant radical scavenging activity against the DPPH and ABTS^+^ with the IC_50_ values of 4.9 and 9.63 μg/ml, respectively	Islam et al. (2020)
**Anti-hyperglycemic activity**						
	REAC	*In vivo*	STZ-induced diabetic mice	150, 300, 600, and 1,200 mg/kg, daily for 21 days	Blood glucose, TC, TGs, and FFAs levels ↓; insulin level ↑; caspase-3, caspase-8, caspase-9, and Bax protein expressions ↓; Bcl-2 protein expression ↑	Xu et al. (2014)
	REAC	*In vivo*	STZ-induced diabetic mice	300, 600, and 1,200 mg/kg, i.g., daily for 14 days	FBG, IL-6 and TNF-α levels ↓; TLR4 and NF-κB mRNA and protein expression	Xu et al. (2015)
	REAC	*In vivo*	STZ-induced diabetic mice	300, 600, and 1,200 mg/kg, i.g., daily for 42 days	FBG, Cr, BUN, and MDA levels ↓; SOD, GSH-Px, CAT activities ↑; Cyto-C, AIF, and caspase-3 protein expressions ↓	Xu et al. (2017)
	FPAC	*In vivo*	Fluoride-induced hyperglycemia, hypercholesterolemia, and oxidative stress of rat’s model	2.5, 5.0, and 10 g, i.g., for 30 days	Blood glucose, G-6-pase, SGOT, SGPT, ACP, and ALP levels ↓; hepatic glycogen and hexokinase, and FRAP activities ↑; plasma AIP, TL, TC, TG, LDL-C, VLDL-C, hepatic lipids-TL, TC and TG levels ↓; HDL-C level ↑; CAT, SOD, GPx, GSH, TAA activities ↑; MDA level ↓	Vasant and Narasimhacharya (2014)
	JEAC	*In vivo*	STZ-induced diabetic mice	25, 50, and 100 mg/kg, i.g., once a day for 21 days	FBG, FFA, TC, TG, Scr, BUN, and MDA levels ↓; SDH, cAMP, SOD, and insulin activities ↑; CTGF and TGF-β1 mRNA and protein expressions ↓	Pham et al. (2017)
	JEAC	*In vivo*	STZ-induced diabetic mice	5, 10, and 20 g/kg, orally, once a day for 14 days	FBG, blood glucose, area under curve, LDH, GC and pyruvate ↓; FINS level ↑	Yang et al. (2019a)
	TFACL	*In vivo*	Alloxan-induced diabetic mice and STZ-induced diabetic rats	0.2, 0.4, and 0.8 g/kg, i.g., daily for 7 days	FBG level ↓; glucose tolerance ↑	Liu et al. (2013)
	EEACB	*In vivo*	STZ-induced diabetic mice	50 and 100 mg/kg, i.g., for three successive days	Blood glucose level ↓	Islam et al. (2020)
	47	*In vivo*	STZ-induced diabetic rats	20 and 50 mg/kg, i.g., for 3 h	Serum blood glucose level ↓; insulin secretion ↑	Cazarolli et al. (2012)
	48					
	48	*In vitro*	^14^C-glucose uptake in rat soleus muscle	50 and 100 μM for 1 h	Glucose uptake and glucose transport ↑	Cazarolli et al. (2009)
	105	*In vivo*	STZ-induced diabetic mice	20, 40, and 80 mg/kg, i.g., daily for 14 days	FBG, FINS, and ISI levels ↓; NF-κB, caspase-3, caspase-8, caspase-9, and Bax protein expressions ↓	Wen et al. (2013)
	106					
	121	*In vivo*	STZ-induced diabetic mice	30, 60, and 120 mg/kg, i.g., once daily, for 21 days	FBG, TC, TG, FFA, GHb, FINS, MCP-1, TNF-α, IL-6 and MDA levels ↓; SOD, GSH activities ↑	Qin et al. (2019)
	121	*In vivo*	STZ-induced diabetic mice	30, 60, and 120 mg/kg, i.g., once daily, for 21 days	TLR4, MyD88, p-NF-κB, TNF-α, and IL-6 mRNA and protein expression levels ↓	Qin et al. (2020)
	122	*In vivo*	Type 2 diabetic KKAy mice	12.5, 25, and 50 mg/kg, i.g., daily, for 56 days	FBG, AGEs glycosylated protein and TC levels ↓; albumin level ↑	Zheng et al. (2013)
	122	*In vivo*	Type 2 diabetic KKAy mice	12.5, 25, and 50 mg/kg, i.g., daily, for 56 days	FBG level ↓; RAGE, NF-B, TGF-β1 and CML protein expression levels ↓	Zheng et al. (2021)
	122	*In vivo*	DN model established by STZ in TLR4 knockout mice and wild-type mice	12.5, 25, and 50 mg/kg, i.g., daily for 28 days	TC, TG, HDL, LDL, Scr, BUN, and blood glucose ↓; IL-6 and TNF-α level ↓; TLR4, MyD88 and NF-κB mRNA and protein expressions ↓	Lu et al. (2019)
	122	*In vitro*	HG-induced HK-2 cells	30 μM for 48 h	Blood glucose ↓; Vimentin mRNA and protein level ↑; TLR4 and E-cadherin mRNA and protein levels ↓; BAMBI ↑; Smad2/3 ↓	Zhang et al. (2019)
	122	*In vivo*	Diabetic kidney disease mice model induced by Wild type and TLR4 knockout	12.5, 25, and 50 mg/kg, i.g., once daily, for 28 days	TC, TG, LDL-C, FBG, CysC, and urinary albumin levels ↓; TLR4, TGF-β1 and Smad2/3 mRNA and protein levels ↓	Zhang et al. (2020)
**Antihyperlipidemic activity**						
	IFRF	*In vivo*	Murine model	Diets formulations, i.g., for 30 days	TG, TC, HDL, and LDL levels ↓	Herman-Lara et al. (2014)
	FF					
	MEACL	*In vivo*	Poloxamer-407-induced hyperlipidemic rat model	NM	TC, TG, LDL-C, VLDL-C and AI levels ↓	Saghir et al. (2016)
	MEACL	*In vivo*	HFD-induced hyperlipidemic rats	250, 500, and 1,000 mg/kg, i.g., daily, for 35 days	TC, TG, LDL-C, VLDL-C, and AI ↓; HDL-C ↑; GSH, GPx, SOD, CAT activities ↑; MDA level ↓	Aladaileh et al. (2019)
**Anti-obesity activity**						
	CEPAC	*In vitro*	3T3-L1 preadipocytes	10, 100, 500, and 1,000 mg/ml	TG accumulation ↓; PPAR-γ and C/EBPα mRNA expressions ↓; PPAR-α mRNA expression ↑	Rashid et al. (2016)
	122	*In vivo*	high-fat diet (HFD) in mice	12.5, 25, and 50 mg/kg, i.g., daily for 28 days	BW and adipose tissue weights, blood glucose, insulin, TC, TG, FFA, IL-6, TNF-α levels ↓; TLR4 and MyD88 expressions ↓; insulin secretion ↑	Li et al. (2016)
**Antitumor activity**						
	ACE	*In vivo*	DENA-induced and CCl4-promoted liver cancer in mice	25 mg/kg, i.g., for five consecutive days	Tumor incidence, tumor yield, tumor burden ↓; LPO level ↓; GSH, SOD, CAT, total proteins content activities ↑	Singh et al. (2014)
	MEACL	*In vivo*	EAC cell bearing mice	25 and 50 mg/kg, i.g., for 5 days	viable cells and body weight ↓; survival time ↑; Hgb, WBC, RBC numbers ↑; p53 and Bax protein expression ↑	Siddika et al. (2020)
	122	*In vitro*	Human breast cancer MCF-7 and BT20 cells	10, 32, 100 μM for 24 h	Caspase-3/7, -8, and -9 activities ↑; TRAIL-R1, TRAIL-R2, Bad, and BID protein expressions ↑; cIAP, XIAP, and Survivin protein expressions ↓; G1 phase cell cycle arrest, ROS ↑; NF-κB ↓	Gao et al. (2015)
	122	*In vivo*	Transplanted 4T1 breast cancer cells bearing mouse	25, 50 and 100 mg/kg, i.g., for 14 days	Survival time ↑; tumor growth ↓; TNF-α, IL-6, IL-12, TGF-β, VEGF ↓; Bax, cleaved caspases-3 and -9 ↑; Bcl-2, MMP-2 and -9, NF-κB and IκBα ↓	Chen et al. (2017b)
	122	*In vitro*	Radio-sensitivity of 4T1 breast carcinoma cell lines	100 μM for 2 or 24 h	TIE, TRD ↓; radio-sensitivity of the 4T1 cells ↑	Muhammed et al. (2019)
	122	*In vitro*	Lung cancer H1299 cells	4.0, 6.0, and 8.0 μg/ml for 24 h	Cell apoptosis ↑; ERK/MAPK ↓; inhibition rates were 22.50, 30.13, and 58.87%, respectively	Zhou et al. (2019)
	122	*In vivo*	Hepatocarcinoma in nude mice	25, 50, and 100 mg/kg, i.g., daily for 12 days	Tumor weight ↓; liver and spleen indexes ↓; IL-2 and IL-10 levels ↓; inhibition rates were 66.39, 63.11, and 47.33%, respectively; WBC, HGB, and PLT numbers ↑; TLR4, MyD88, and NF-κB expressions ↓	Wu et al. (2020a)
**Anti-inflammatory activity**						
	EEACL	*In vivo*	Croton oil-induced mouse ear edema	0.03–1.0 mg/ear	Edema (IC_50_: 0.05) ↓; MPO activity (IC_50_: 0.22) ↓	Cabrini et al. (2011)
	PFSCW	*In vivo*	Adult female Swiss mice	100 and 300 mg/kg	Paw edema ↓	Leivas et al. (2016a)
	122	*In vivo*	Pancreatic β-cell line Min6 cells	10, 15, and 20 μmol/L	TNF-α, IL-6 and MCP-1 level ↓; cleaved-caspase-3, -8 and -9, TLR4, MyD88, and NF-KB expressions ↓; Bcl-2/Bax ratio ↑	Xie et al. (2016)
**Hepatoprotective activity**						
	FPEAC	*In vivo*	Leptin receptor- deficient (db/db) mice	10, 20, and 30 g/kg, i.g., daily, for 8 weeks	TG, TC, LDL-C, and NEFA level ↓; AST and ALT activities ↓; HDL-C level ↑; p-AMPK protein expression ↑; SREBP-1c, FAS and SCD1 mRNA and protein expression levels ↓; mircoRNA-34a and mircoRNA-33 expression levels ↓	Pang et al. (2017)
	FJAC	*In vivo*	STZ-induced diabetic mice	5, 10, and 20 g/kg, i.g., daily for 14 days	MAD and cAMP levels ↓; SDH, MDA, and SOD activities ↑	Yang et al. (2018)
	EACR	*In vivo*	CCl_4_-induced acute liver injury in mice	0.3, 0.6, and 1.2 g/kg, i.g., dialy, for 7 days	f AST, ALT, IL-1, IL-6, MDA levels ↓; SOD, GSH, GSH-Px activities ↑; TNF-α, NF-κB, caspase-3 protein expression levels ↑	Huang et al. (2019)
	EACR	*In vivo*	Liver fibrosis (HF) rats induced by CCl_4_	0.25, 0.5, and 1.0 g/kg, i.g., dialy, for 28 days	Albumin/globulin (A/G) ratio ↑; TBIL and TC levels ↓; NF-κB and Bax expression levels ↓; Bcl-2 expression level ↑	Liang et al. (2020b)
	EACR	*In vivo*	CCl_4_-induced acute liver injury in rats	0.25, 0.5, and 1.0 g/kg, i.g., dialy, for 8 weeks	AST, ALT, AKP, Hyp, HA, LN, Col III, Col IV, MDA levels ↓; SOD and GSH-Px activities ↑; COL-1a1, α-SMA, TIMP2, TGF-β1, Smad-2 and Smad-4 mRNA expression levels ↓; α-SMA, TIMP2, TGF-β1, Smad-2, Smad-3 and Smad-4, Bax and cleaved caspase-3 proteins expression levels ↓; Smad-7 mRNA expression and Smad-7 and Bcl-2 protein expression ↑	Huang et al. (2020)
**Cardioprotective activity**						
	AEAC	*In vivo*	Rats with ventricular remodelling induced by isoprenaline	50, 100, and 200 mg/kg, i.g., daily, for 14 days	TGF-β, Ang II, iNOS, ECE, ET-1 levels and expressions ↓; VR index, CVF ↓; tNOS, eNOS protein expression levels ↑	Liang et al. (2020a)
**Antihypertensive activity**						
	AELAC	*In vivo*	Normotensive rats	12.5–50.0 mg/kg, i.v.	MAP ↓; Ca^2+^-free medium ↓	Soncini et al. (2011)
	EEACR	*In vivo*	Normal rats	150, 300, and 600 mg/kg, i.g.	Blood pressure ↓	Tang et al. (2017)
	FACF	*In vivo*	Normal rats and rats with hypertension induced by L-NAME	300, 600, and 1,200 mg/kg, i.g., daily, for 5 weeks	SBP, DBP, MBP, blood pressure ↓	Huang et al. (2017)
**Neuroprotective activity**						
	122	*In vivo*	APP/PS1 transgenic AD mice	12.5, 25, and 50 mg/kg, i.g., once a day for 21 days	Spatial learning and memory deficit, fear memory deficit apoptosis and loss of neuron ↑; Bcl-2/Bax ratio ↑	Wei et al. (2018)
	122	*In vitro*	PC-12 cells	5, 10, and 20 μmol/L	Bcl-2 mRNA and protein expressions ↑; Bax mRNA and protein expressions ↓; caspase-3 and caspase-9 expressions ↑; Bcl-2/Bax ratio ↑	Wei et al. (2018)
	122	*In vitro*	SH-SY5Y cells induced by Aβ1-42	5, 10, and 20 μmol/L	cell viability loss and apoptosis ↓; Bax, caspase-3, caspase-8 and caspase-9 protein expression levels ↓; Bcl-2 protein expression ↑	Lu et al. (2020)
**Reducing UVB-induced skin damage**						
	EFAC	*In vitro*	Human HaCaT keratinocytes induced by UVB	50, 100, and 250 µg/ml	Apoptotic cells number ↓; caspase 3 expression ↓; CPD ↓	Ronpirin et al. (2016)
	AFAC					

NM, not mentioned; ACL, *A. carambola* leaves; MEACL, methanol extract of *A. carambola* leaves; EEBAC, ethanol extracts of bark from *A. carambola*; REAC, root extracts of *A. carambola*; FPAC, fruit powder of *A. carambola*; JEAC, juice extracts of *A. carambola*; TFACL, total flavones from *A. carambola* leaf; EEACB, ethanol extracts of *A. carambola* bark; MEACL, methanolic extract of *A. carambola* leaves; CEPAC, crude extract from peel of *A. carambola*; PEFAC, a homogenous polysaccharide extracted from the fruit of *A. carambola*; ACE, *A. carambola* extracts; EEACL, ethanol extract of *A. carambola* leaves; FPEAC, free phenolic extract from *A. carambola*; FJAC, fruit juice of *A. carambola*; EACR, extract of *A. carambola* root; AEAC, aqueous extract of *A. carambola*; AELAC, aqueous extract of leaves of *A. carambola*; EEACR, ethanol extracts of *A. carambola* root; FACF, flavonoids in *A. carambola* fruit; EFAC, ethanol fractions of *A. carambola*; AFAC, aqueous fractions of *A. carambola*; 40, Norathyriol; 47, Carambolaflavone A; 48, Carambolaflavone B; 105, (+)-lyoniresinol 3α-O-β-D-glucopyranoside; 106, (−)-lyoniresinol 3α-O-β-D-glucopyranoside; 121, 2-methoxy-6-nonyl-cyclohexa-2,5-diene-1,4-dione; 122, 2-dodecyl-6-methoxycyclohexa-2,5-diene-1,4-dione.

### Anti-Hyperglycemic Activity

Diabetes mellitus is characterized by a chronic hyperglycemic condition possibly induced by insulin deficiency, damage to insulin signaling, or non-autoimmune etiology or caused by a remarkably diminished insulin sensitivity (Teng et al., 2018). This disorder poses a significant public health care problem, with its prevalence continuing to rise globally (Chen et al., 2019). Type 2 diabetes mellitus, in particular, is primarily distinguished by insulin resistance and β cell dysfunction, which cause insulin secretion reduction (Uuh-Narváez et al., 2021). Diabetes mellitus patients can die if they develop diabetic kidney disease.

**FIGURE 4:**
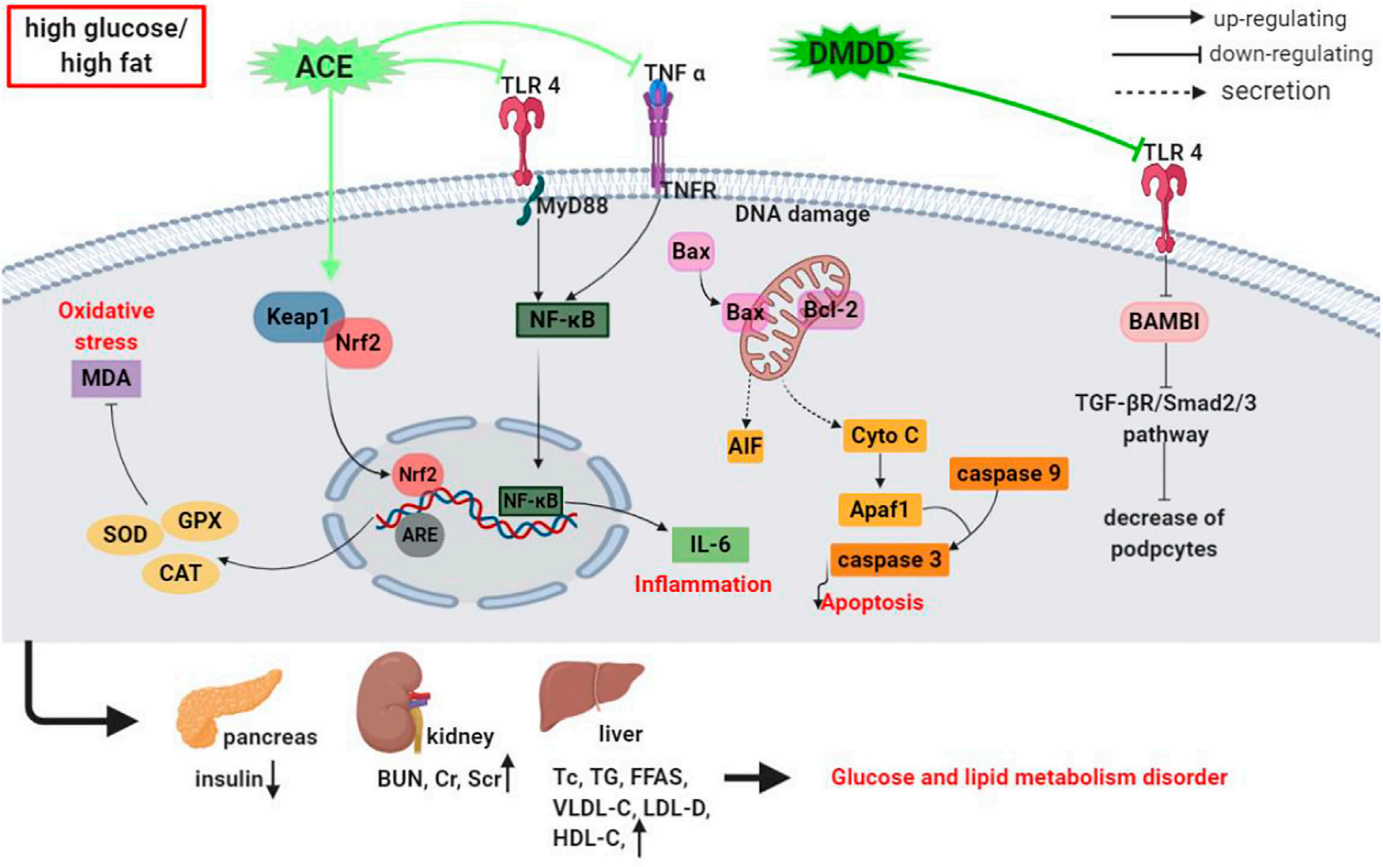
Schematic representation of the underlying mechanism of anti-hyperglycemic activity of crude extracts or isolated compounds from *A. carambola*.

In the past 5 years, several studies have extensively explored the antidiabetic potential of *A. carambola* using various experimental models. The findings have revealed that *A. carambola* and its glycosides exhibit outstanding antidiabetic features, and insights into the underlying mechanisms have also been provided, although they are not yet fully understood. The underlying mechanism of the anti-hyperglycemic activity of crude extracts or bioactive substances from *A. carambola* is presented in Table 2 and Figure 4.

#### Crude Extract

Evaluations of the hypoglycemic activity of *A. carambola* crude extracts on rats and mice have been conducted with encouraging results. In one such assessment, mice with STZ-induced diabetes given root extracts of *A. carambola* (REAC at daily doses of 150, 300, 600, and 1,200 mg/kg for 21 days) orally had significantly decreased blood glucose, TC, TGs, and FFAs levels and elevated insulin content in their serum. Mechanically, REAC markedly downregulated pro-apoptosis caspase-3/8/9 and Bax protein expressions and upregulated anti-apoptotic Bcl-2 protein expression. Additionally, REAC prevented pancreatic β cell apoptosis in these mice (Xu et al., 2014). The findings of that investigation suggest that REAC possesses remarkable hyperglycemic abilities that could improve metabolic functions and suppress the apoptosis of pancreatic β cells.

Research into the anti-hyperglycemia activities of REAC on regulating the TLR4/NF-κB signaling pathway in STZ-induced diabetic mice showed that REAC (at daily doses of 300, 600, and 1,200 mg/kg, i.g. for 14 days) decreased the serum contents of fasting blood glucose (FBG), TNF-α, and IL-6 significantly and downregulated TLR4 and NF-κB protein and mRNA expressions in pancreatic tissue (Xu et al., 2015). In their assessment of the protective features of REAC on renal function injury in STZ-stimulated diabetic mice, Xu et al. (2017) found that REAC treatment reduced FBG, blood urea nitrogen (BUN), and creatinine levels in serum significantly, strengthened SOD, GSH-Px, and CAT activities considerably, and lessened MDA levels and Cyto-C, AIF, and caspase-3 protein expressions in the kidney tissues of the mice.

After orally treating fluoride-induced hyperglycemia, hypercholesterolemia, and oxidative stress in rats with fruit powder from *A. carambola* (FPAC, at daily doses of 2.5, 5.0, and 10 g for 30 days), the plasma glucose level and G-6-pase, SGOT, SGPT, ACP, and ALP activities reduced notably, while the activities of hepatic glycogen, hexokinase, and FPAC increased dose-dependently. Increments in the atherogenic index of plasma (AIP), total lipids (TL), TC, TG, LDL-C, and VLDL-C in plasma, TL, TC, and TG in the liver and the decrease in HDL-C were also reversed. Additionally, FPAC prompted an increase in CAT, SOD, GSH-Px, GSH, and total ascorbic acid (TAA) activities significantly, and a decline in MDA content in the hepatic and renal tissues of rats (Vasant and Narasimhacharya, 2014).

STZ-induced diabetic mice receiving juice extracts from *A. carambola* (JEAC) intragastrically (at daily doses of 25, 50, and 100 mg/kg for 21 days) have displayed markedly reduced FBG, FFA, MDA, TC, TG, serum creatinine (Scr), and BUN levels and significantly increased insulin, sorbitol dehydrogenase (SDH), cAMP, and SOD activities. In the same study, diabetes-instigated changes in kidney tissues, including thickened and tubular basement membranes and glomerular hypertrophy, greatly improved, and the expressions of related mRNAs and proteins, such as CTGF and TGF-β1, in kidney tissues markedly decreased after JEAC treatment (Pham et al., 2017). JEAC (at daily doses of 5, 10, and 20 g/kg, i.g. for 14 days) has also been proven to considerably decrease FBG levels, blood glucose, area under the curve, LDH, glucagon (GC), and pyruvate in serum and increase fasting insulin (FINS) levels in STZ-induced diabetic mice, indicating that this extract could lessen hyperglycemia and hyperlipidemia, suppressing DN progression and development; it is a potential candidate agent for treating or preventing DN (Yang et al., 2019a).

Liu et al. (2013) reported that the total flavones from *A. carambola* leaf (TFACL at daily doses of 0.2, 0.4, and 0.8 g/kg, i.g. for 7 days) significantly lowered FBG levels and enhanced glucose tolerance in mice and rats with diabetes mellitus. The STZ-induced diabetic mice also received ethanol extracts of *A. carambola* bark (EEACB) orally (at daily doses of 50 and 100 mg/kg for three successive days), which drastically decreased their level of blood glucose 150 min after orally receiving a single dose of glucose solution (1.0 g/kg); 100 mg/kg EEACB triggered a higher blood glucose level decline than 50 mg/kg EEACB (Islam et al., 2020). These results suggest that *A. carambola* is a potential hypoglycemic drug for the prevention and treatment of diabetes.

#### 6.2.2 Isolated Compounds

As previously mentioned, some compounds, including **47**, **48**, **100**, **101**, **120,** and **121**, isolated and identified from *A. carambola* have been tested for their potential anti-hyperglycemic activity *in vivo*. Normal hyperglycemic rats receiving an oral treatment of flavonoid carambolaflavone A (at doses of 20 and 50 mg/kg) **(47)** and carambolaflavone B (at doses of 20 and 50 mg/kg) **(48)** extracted from *A. carambola* leaves demonstrated decreased glycemia levels, suggesting a potential hypoglycemic effect. Glycogen levels in the muscle and liver also increased sharply (Cazarolli et al., 2012). Another study by the same authors also found 50 mg/kg of carambolaflavone B **(48)** to notably decrease blood glucose levels in diabetic rats and provoke glucose-triggered insulin secretion after oral treatment of the hyperglycemic rats. Additionally, a noteworthy stimulatory function of compound **48** (at concentrations of 50 and 100 μM) on ^14^C-glucose uptake was observed, but treatment with inhibitors, including wortmannin, RO318220, and PD98059, reversed this activity. Interestingly, 100 μM of compound **48** and 10 nM of insulin had no synergistic effect on glucose uptake (Cazarolli et al., 2009).

Chiral lignan glucosides, (±)-lyoniresinol 3α-O-β-D-glucopyranoside (LGP1 **(105)** and LGP2 **(106)**), isolated from *A. carambola* root have been evaluated for health-promoting properties in LGP1 and LGP2-mediated hypoglycemia against renal injury in STZ-created diabetic mice. Diabetic mice that received LGP1 and LGP2 (at daily doses of 20, 40, and 80 mg/kg for 14 days) yielded contrasting outcomes. While LGP1 markedly alleviated the histopathological changes in the kidney and lowered FBG, FINS, and insulin sensitivity index (ISI) levels and caspase-3/8/9, Bax, and NF-κB protein expressions, LGP2 had no impact (Wen et al., 2013), suggesting that LGP1 could attenuate and treat the progression of DN through regulating several molecular targets.

Benzoquinone (**121**) isolated from the roots of *A. carambola* has also been analyzed, and, at doses of 30, 60, and 120 mg/kg, i.g. once a day for 21 days. It effectively decreased serum FBG, TC, TG, FFA, MCP-1, TNF-α, GHb, FINS, MDA, and IL-6 levels and increased SOD and GSH activities in STZ-induced diabetic rats. Mechanistically, the extract strikingly downregulated TNF-α, IL-6, MyD88, p-NF-κB, and TLR4 expressions in pancreatic tissues (Qin et al., 2019; Qin et al., 2020). These findings suggest that benzoquinone (**121**) exerts a protective effect against STZ-induced hyperglycemia, and the underlying molecular mechanism of its impact could be associated with the suppression of the activation of the TLR4/NF-κB signaling pathway.

Zheng et al. (2013), Zheng et al. (2021) reported that DMDD **(122)** isolated from *A. carambola* dry roots (at daily doses of 12.5, 25, and 50 mg/kg, i.g. for 56 days) to significantly reduce FBG, creatinine clearance, proteinuria, Scr, and serum urea-N contents and glomerular mesangial matrix expansion. It also markedly improved renal AGE formation, decreased AGE receptor, NF-κB, TGF-β1, and N^ε^-(carboxymethyl)lysine expressions in diabetic mice, and effectively alleviated DN in type 2 diabetic KKAy mice. However, the underlying mechanism of how DMDD alleviates DN has yet to be demonstrated; therefore, the precise action mechanism of DMDD against DN must be explored further (Lu et al., 2019). STZ-established DN in TLR4 knockout (TLR4^−/−^, KO) and wild-type (WT) mice treated orally with DMDD (at doses of 12.5, 25, and 50 mg/kg for 28 days) lowered serum TC, TG, HDL-C, and LDL-C, blood glucose content and kidney function markers, including Scr and BUN, significantly and increased the quantity and density of podocytes spectacularly, contributing to DN symptom alleviation. The treatment also markedly inhibited IL-6 and TNF-α levels and blocked the TLR4/MyD88/NF-κB signaling pathways. These effects, though, were notably different in TLR4^−/−^ mice. *In vitro*, 30 μM DMDD radically subdued TLR4, Smad2, and Smad3 expressions in high glucose (HG)-stimulated HK-2 cells and diminished BMP and activin membrane-bound inhibitor (BAMBI) expressions (Zhang et al., 2019). Additionally, the increase in Smad2/3 expression and decrease in BAMBI expression in HG cultured cells were markedly annulled in cells treated with TAK-242 (a TLR4 signaling inhibitor). More importantly, BAMBI gene silencing extraordinarily enhanced the epithelial-mesenchymal transition (EMT) process and its over-expression did the opposite in HK-2 cells under HG conditions. EMT was eased with the pre-administration of DMDD to HK-2 cells, pointing to DMDD’s protective effects on HG-induced EMT in HK-2 cells *via* suppressing the TLR4/BAMBI/Smad2/3 signaling pathways (Zhang et al., 2019). These findings insinuate that DMDD isolated from *A. carambola* is a potential therapeutic drug for DN treatment.

In Zhang et al. (2020) study of the antidiabetic activity of DMDD isolated from *A. carambola* roots in Wild type and TLR4 knockout mice with diabetic kidney disease, DMDD (at daily doses of 12.5, 25, and 50 mg/kg, i.g. for 28 days) caused notable reductions in urinary albumin, TC, TG, LDL-C, FBG, and CysC levels. The extract also alleviated pathological changes and renal fibrosis clearly but silencing the TLR4 gene improved the pathology. Mechanistically, TLR4, TGF-β1, Smad2, and Smad3 protein expressions in renal tissues decreased pointedly after DMDD treatment. TGF-β1 and Smad2/3 genes and protein expressions also unusually declined in TLR4^−/−^ mice than Wild type mice. Furthermore, according to IHC results, there were strong *in situ* expressions of TLR4, TGF-β1, Smad2, and Smad3 in kidney tissues, which were distinctly weakened after DMDD-treatment. Nevertheless, TGF-β1, Smad2, and Smad3 levels did not increase in the TLR4^−/−^ mice with significance when compared with healthy mice (Zhang et al., 2020). These findings strongly suggest that TLR4 is critical for DMDD’s protective activity against renal insufficiency in diabetic mice, and hypoglycemic and anti-fibrosis properties could be regulated by the TLR4/TGF signaling pathway.

In summary, *A. carambola* could attenuate oxidative injury and inflammatory response in rat models by enhancing antioxidant enzyme activities and decreasing inflammatory cytokine levels, which result in insulin secretion, as well as blood glucose and blood lipid metabolism regulation. Some original findings on active compounds and their related mechanisms also exist; however, these investigations lack clear indications of whether these activities stem from a single substance or synergistic effects of several substances of *A. carambola*; therefore, before they can be advanced as bases for clinical research and exploration of the medicinal values of *A. carambola*, further evaluations must be performed.

### Anti-Hyperlipidemic Activity

Cardiovascular diseases (CVDs) are among the most common causes of death globally, with hyperlipidemia representing one of its main risk factors (Mahdavi et al., 2020; Jayachandran and Qu, 2021). In Herman-Lara et al. (2014) evaluation of the protective activity of micronized insoluble fiber from the bagasse of *A. carambola* as an ingredient of a functional food (FF) or as micronized insoluble fiber-rich fraction (IFRF) and its effects *in vivo* on lipid metabolism in a murine model, serum TG, TC, HDL-C, and LDL-C decreased after a murine model was treated with IFRF and FF, with corresponding inhibition rates of 14.2, 25.4, 55.06, and 12.18% by IFRF, and 30.18, 39.47, 35.11, and 43.18% by FF. IFRF treatment also produced higher hypolipidemic activity and greatly avoided the occurrence of non-alcoholic fatty liver. Overall, IFRF and FF exerted hypolipidemic qualities, indicating that the star fruit’s insoluble fiber is potentially a component of FFs in the treatment and prevention of CVDs (Herman-Lara et al., 2014).

In Saghir et al. (2016) investigation of the anti-hyperlipidemic activity of methanolic and aqueous extracts from various *A. carambola* parts (such as leaves, stems, and ripe and unripe fruits), they found that the methanolic extract of *A. carambola* leaves (MEACL) had the most potent hypolipidemic effect in poloxamer-407-induced hyperlipidemic rats and effectively decreased serum TC, TG, LDL-C, VLDL-C, and atherogenic index (AI) levels. MEACL (at daily doses of 250, 500, and 1,000 mg/kg, i.g. for 35 days) significantly reduced serum and liver lipids levels, including TC, TG, LDL-C, VLDL-C, and AI, elevated HDL-C, and dose-dependently alleviated histopathological changes in the liver of HFD-induced hyperlipidemic rats. MEACL also considerably enhanced GSH, GSH-Px, SOD, and CAT activities and lowered MDA levels. Furthermore, MEACL treatment inhibited HMG-CoA reductase and lipase (Aladaileh et al., 2019). These results demonstrate that MEACL has a hypolipidemic activity it could exert by ameliorating lipid peroxidation and improving antioxidant defenses in HFD-fed rats (Table 2).

### Anti-Obesity Activity

Obesity-related diseases are increasingly becoming common public health and social issues. They can cause a multitude of metabolic disorders closely associated with increased risk of many chronic diseases (Zhu et al., 2015; Li et al., 2016). One study found that some phytochemicals suppressed adipogenesis and obesity. According to Rashid et al. (2016), the crude extract from the peel of *A. carambola* (CEPAC) (at concentrations of 10, 100, 500, and 1,000 mg/ml) dose-dependently inhibited TG accumulation in their research. Additionally, 1,000 mg/ml of CEPAC substantially blocked most of the adipocyte differentiation in 3T3-L1 preadipocytes with no toxicity. The extract also notably reduced the mRNA expressions of two master adipogenic transcription factors, PPAR-γ and C/EBPα, and increased the PPAR-α receptor at the molecular level. In further phytochemical analyses, the authors recognized (-)-epicatechin as a natural bioactive molecule responsible for the features presented above (Rashid et al., 2016). These findings indicate that CEPAC is potentially promising in treating obesity and obesity-related disorders.

Per Li et al. (2016) inquiry into the benefits of DMDD against high-fat diet (HFD)-evoked obesity and insulin resistance in mice, DMDD (at daily doses of 12.5, 25, and 50 mg/kg, i.g. for 28 days) markedly decreased body and adipose tissue weights and reduced insulin, blood glucose, TC, TG, FFA, IL-6, and TNF-α levels in serum. DMDD also significantly downregulated TLR4 and MyD88 protein levels in epididymal adipose tissues, considerably enhanced insulin secretion, lowered the areas under the curve, notably heightened SOD and GSH-Px activities, and decreased MDA content in mice liver tissues (Li et al., 2016). Based on these results, DMDD possesses potential benefits that could be used to treat HFD-induced obesity and insulin resistance, potentially improving the lipid metabolism process and the suppression of TLR4 protein expression in adipose tissues (Table 2).

### Antitumor Activity

**FIGURE 5:**
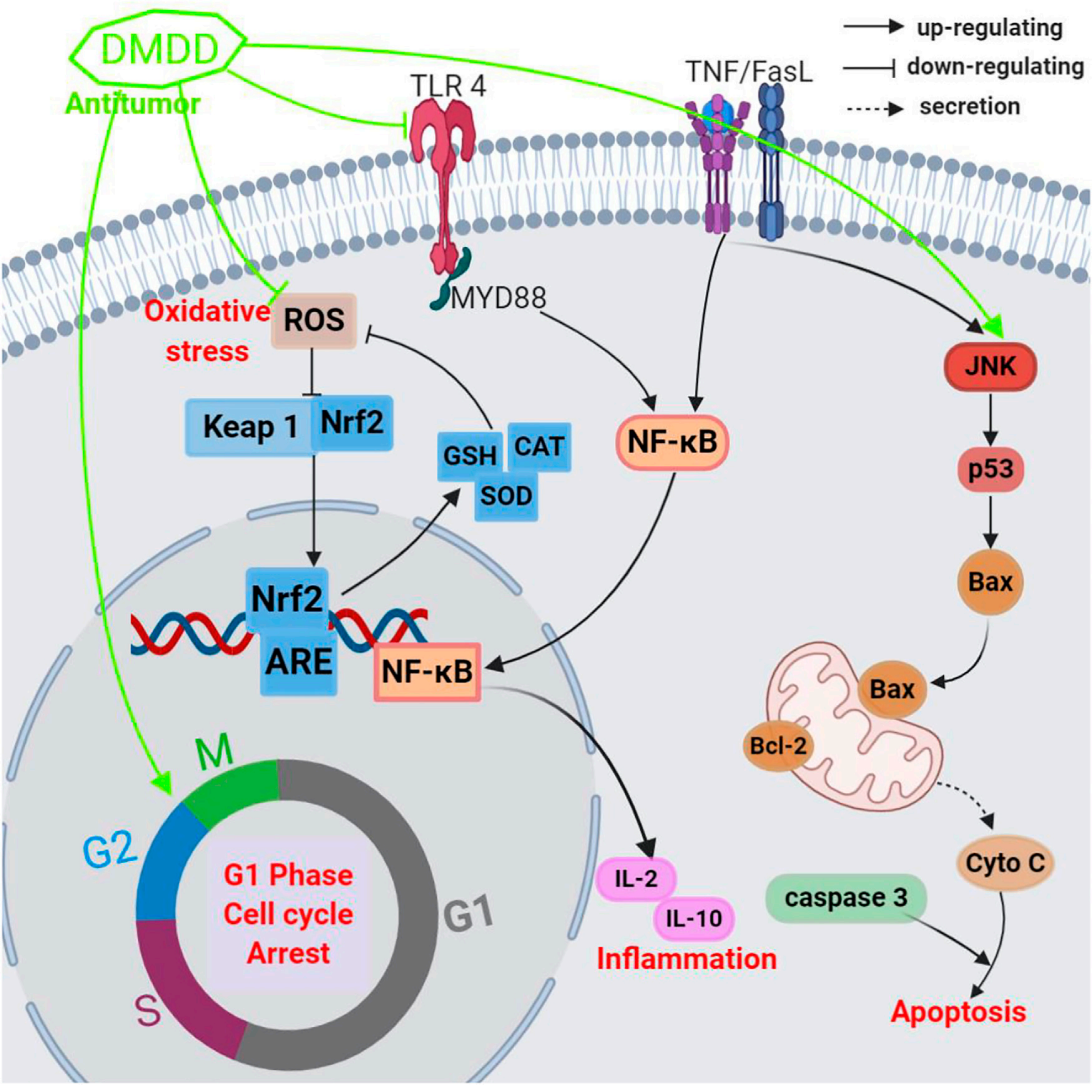
Schematic representation of the underlying mechanism of anti-tumor activity of crude extracts or isolated compounds from *A. carambola*.

Several studies have demonstrated the significant inhibitory activity of *A. carambola* against a variety of tumor cells *in vivo* and *in vitro* and the possible mechanism of the antitumor activity of crude extracts or isolated compounds from *A. carambola* (Table 2 and Figure 5). DENA-induced and CCl_4_-promoted liver cancer mice given *A. carambola* extracts (ACE, at a dose of 25 mg/kg for 5 days) orally resulted in ACE considerably lowering tumor incidence, tumor yield, and tumor burden. The treatment also significantly reduced lipid peroxidation (LPO) levels and elevated GSH, SOD, CAT activities (Singh et al., 2014). These findings hinted at ACE’s ability to exert a protective effect against liver cancer in mice and could be used as a good natural chemo-preventive product against cancer to further screen active components. Supposedly, MEACL (25 and 50 mg/kg, i.g. once a day for 5 days) significantly reduces body weight and cell viability, prolongs survival time, and improves altered hematological parameters (HGB, WBC, RBC) in Ehrlich ascites carcinoma (EAC) cell-bearing mice. MELA could induce cancer cell apoptosis through upregulating p53 and Bax protein expressions at the molecular level (Siddika et al., 2020).

Gao et al. (2015) also showed that DMDD significantly suppressed breast cancer MCF-7 and BT20 cell growth, with the IC_50_ values ranging from 3.13 to 5.57 μM; DMDD exerted anticancer traits by inducing apoptosis and blocking the cell cycle at the G_1_ phase, increasing the generation of intracellular ROS, which activated the extrinsic receptor and intrinsic mitochondrial pathways and inhibited NF-κB activation. One study using 4T1 breast cancer cell-bearing mice for *in vivo* research revealed that DMDD radically inhibited primary breast tumor growth and suppressed breast tumor metastasis in the lung and liver, as well as decreased inflammatory cytokine production, induced cell apoptosis, and prevented the activation of the TNF-α/NF-κB/MMPs pathways, prolonging the survival time of tumor-bearing mice (Chen et al., 2017b). These outcomes imply that DMDD has potent antitumor features that could help treat metastatic breast cancer.

Muhammed et al. (2019) explored the radio-sensitivity of 4T1 tumor cells treated with DMDD extracted from *A. carambola* roots, by analyzing the TIE and TRD of the *in vivo* 2 and 24 h treatment groups compared with the untreated control group. The threshold ionization energy (TIE) and threshold radiation dose (TRD) was determined utilizing a novel method that employs a laser trapping technique for single and multiple cell ionizations. Per the findings, treatment periods increased, and TIE and TRD notably decreased, pointing to DMDD’s powerful enhancement effect on 4T1 cell radio-sensitivity. However, TRD diminished with mass regardless of the intervention. TRD analyses of single vs multiple cells ionizations within each group continuously demonstrated the same behavior even after treatment. The underlying elements for these observed relations could be explained in terms of radiation, hyperthermia, and chemo effects (Muhammed et al., 2019). DMDD treatment (at 4.0, 6.0, and 8.0 μg/ml for 24 h) has been shown to dramatically and dose-dependently inhibit the proliferation, migration, and invasion of H1299 cells, with inhibition rates of 22.50, 30.13, and 58.87%, respectively. The same research determined that DMDD also induced the apoptosis of H1299 cells, with the mechanism possibly associated with the suppression of the ERK/MAPK pathway (Zhou et al., 2019).

Wu et al. (2020a) assessed the antitumor effect of DMDD on hepatocarcinoma in nude mice and its mechanism, the tumor weight in mice decreased significantly, with tumor inhibition rates for the three doses amounting to 66.39, 63.11, and 47.33%, respectively, after daily oral treatment with DMDD at 25, 50, and 100 mg/kg for 12 days. The tumor and organ indexes, including liver and spleen, and IL-2 and IL-10 serum levels also notably decreased, and the amounts of WBC, HGB, and PLT dramatically increased. Additionally, the expression of TLR4, MyD88, and NF-κB reduced markedly. These results suggest that DMDD has promising anticancer properties, with its mechanism of action is possibly linked to the repression of the TLR4/MyD88/NF-κB signaling pathways (Table 2).

### Anti-Inflammatory Activity

The evaluation of the anti-inflammatory effects of the ethanol extract of *A. carambola* leaves (EEACL), its ethyl acetate, butanol, and hexane fractions, and two flavonoids against skin inflammation in mice with croton oil-stimulated ear edema revealed that topically used EEACL (at concentrations of 0.03–1.0 mg/ear) shrunk the edema dose-dependently, with an ID_50_ value of 0.05 at concentrations of 0.02–0.13 mg/ear and maximum inhibition of 73% at a concentration of 0.6 mg/ear. EEACL also suppressed myeloperoxidase (MPO) activity, with an ID_50_ value of 0.22 and a maximum inhibition of 61% at a concentration of 0.6 mg/ear. Furthermore, all the fractions examined markedly curbed edema formation and lowered MPO activity. Additional evaluations found that the ethyl acetate fraction of EEACL was the most effective inhibitor of MPO activity and edema formation, with inhibition rates of 54 and 75%, respectively (Cabrini et al., 2011). Unfortunately, EEACL bioactive substances against skin inflammation have not been tested extensively so far; therefore, they require further scrutiny to be optimized and developed into effective remedies for preventing and treating skin disorders.

GC-MS analyses of a homogenous polysaccharide extracted from the fruit of *A. carambola* (PFSCW, *Mw* 40 kDa) established that it consisted mainly of arabinose, galactose, and galacturonic acid in a molar ratio of 12.3:1.7:86.0. Methylation and NMR spectroscopy examination identified a substituted galacturonan composed of (1→4)-linked-α-D-Galp A units with a branch at O-2 by (1→5)-linked-α-L-Araf and terminal-α-L-Araf and α-D-Galp A units as components of PFSCW. PFSCW treatment (at doses of 100 and 300 mg/kg) drastically reduced intraplantar formalin-injected paw edema, with an inhibition rate of 53% at a dose of 300 mg/kg, suggesting that the extract has moderate anti-inflammatory properties (Leivas et al., 2016a). Xie et al. (2016) demonstrated that DMDD (at concentrations of 10, 15, and 20 μM) significantly inhibited palmitic acid (PA)-stimulated inflammation in pancreatic Min6 cells by strongly obstructing the TLR4/MyD88/NF-KB pathways, which lowered the generation of inflammatory cytokines (TNF-α, IL-6, and MCP-1), downregulating cleaved-caspase-3/8/9 protein expression, elevating the Bcl-2/Bax ratio, and decreasing TLR4, MyD88, and NF-KB protein expressions. These outcomes hint at DMDD's ability to reverse PA-stimulated Min6 cell dysfunction via alleviating cell apoptosis and inflammatory response, with the underlying mechanism of action almost certainly associated with the suppression of the TLR4/MyD88/NF-κB signaling pathways (Table 2).

### Hepatoprotective Activity

An evaluation of the hepatic steatosis alleviating activity of a free phenolic extract of *A. carambola* (FPEAC) in leptin receptor-deficient (db/db) mice to clarify the underlying mechanisms of action for modulating hepatic lipogenesis found that FPEAC (at daily doses of 10, 20, and 30 g/kg, i.g. for 8 weeks) considerably lowered LDL-C, TG, TC, non-esterified fatty acid (NEFA) levels, and AST and ALT activities, elevated serum HDL-C level, and reduced TG content in the liver. Mechanically, FPEAC drastically downregulated sterol regulatory element-binding protein-1c (SREBP-1c), fatty acid synthase (FAS), and stearoyl-CoA desaturase 1 (SCD1) expressions in hepatic tissues and markedly upregulated p-AMPK α levels. More importantly, FPEAC significantly downregulated microRNA-34a and microRNA-33 expressions, which regulate the AMPK/SREBP-1c/FAS signaling pathway (Pang et al., 2017). These findings indicate that FPEAC has a compelling hepatic steatosis mitigating effect, partially through suppressing the signal transmission of hepatic lipogenesis. According to Yang et al. (2018), the fruit juice of *A. carambola* (FJAC, at daily doses of 5, 10, and 20 g/kg, i.g. for 14 days) showed potential hepatoprotective properties; it reduced MAD and cAMP levels and increased SDH, MDA, and SOD activities in the liver of mice with STZ-induced diabetes.

In one study, mice with acute liver injury given the extract of *A. carambola* roots (EACR, at daily doses of 0.3, 0.6, and 1.2 g/kg for 7 days) had significantly lower serum CCl_4_, AST, ALT, IL-1, and IL-6 levels and liver MDA levels but considerably increased SOD, GSH, and GSH-Px activities. At the molecular level, TNF-α, NF-κB, and caspase-3 protein expressions were significantly downregulated in the liver. HE staining showed that the liver injury was eased (Huang et al., 2019). In another investigation, EACR (at daily doses of 0.25, 0.5, and 1.0 g/kg, i.g. for 28 days) substantially increased the albumin/globulin (A/G) ratio, reduced total bilirubin (TBIL) and TC levels in the liver microstructure of rats with CCl_4_-induced liver fibrosis (HF), markedly lessened NF-κB and Bax protein expressions, and considerably augmented Bcl-2 protein expression (Liang et al., 2020b). Rats with CCl_4_-instigated chronic liver injury treated intragastrically with EACR (at doses of 0.25, 0.5, and 1.0 g/kg for 56 days) displayed drastically decreased serum AST, ALT, AKP, and Hyp content, serum hepatic fibrosis biomarkers (HA, LN, Col III, and Col IV), and liver tissue MDA content and increased SOD and GSH-Px capacities. EACR also considerably reversed the elevation in COL-1a1, α-SMA, TIMP2, TGF-β1, and Smad-2 and -4 mRNA expressions and inhibited α-SMA, TIMP2, TGF-β1, Smad-2, -3, -4, Bax, and caspase-3 levels in liver tissues. Additionally, the extract significantly increased Smad-7 mRNA expression and Smad-7 and Bcl-2 protein levels in the liver (Huang et al., 2020). These findings point to EACR being a promising remedy for liver fibrosis. However, further studies must be conducted urgently to identify the bioactive components and possible mechanism of EACR’s anti-fibrotic effects (Table 2).

### Cardioprotective Activity

Ventricular remodeling (VR) results in changes in endothelial vasoactive substances, cardiomyocyte hypertrophy, myocardial fibrosis, and endothelial dysfunction. Per Liang et al. (2020a) investigation of the protective property of the aqueous extract of *A. carambola* (AEAC) on isoprenaline-stimulated endothelial function in rats with VR, AEAC (at daily doses of 50, 100, and 200 mg/kg, i.g. for 14 days) glaringly lowered serum levels of iNOS, TGF-β, Ang II, ECE, and ET-1 and their protein expressions and decreased the VR index and CVF but markedly elevated serum tNOS and eNOS levels and their protein expressions. Pathological evaluations demonstrated that AEAC radically alleviated inflammatory infiltration, apoptosis, fibrosis, and necrosis in rat myocardial tissues, suggesting that AEAC potentially mitigates the VR of rats and is, therefore, possibly associated with safeguarding the balance of vasoactive components and vascular endothelium function (Liang et al., 2020a). Another study showed that the high-glucose-high-fat diet combined with STZ induced the diabetes mellitus mice model administrated with DMDD (at doses of 12.5, 25, and 50, 100 mg/kg/day, i.g., for 21 days) markedly alleviated the myocardial tissues damage, inhibited the myocardial cell apoptosis, reduced the levels of FBG, LVEDP, ROS, MDA, Beclin-1, LC3II/I, and Atg5 as well as increased the SOD, p-PI3K/PI3K, p-Akt/Akt, and p-mTOR/mTOR, indicating that DMDD exerts cardioprotective activity *via* regulating the ROS-mediated PI3K/Akt/mTOR autophagy pathways (Table 2; Ma et al., 2021).

### Anti-Hypertensive Activity

In Soncini et al. (2011) study of the anti-hypertensive effect of the aqueous extract of leaves of *A. carambola* (AELAC) in an isolated rat aorta and its possible mechanism, AELAC treatment (at doses of 12.5–50.0 mg/kg, i.v.) resulted in a remarkable dose-dependent decrease in mean arterial pressure (MAP) in normotensive rats. *In vitro*, AELAC reduced *E*_max_ response to phenylephrine but triggered no sensitivity change. AEAC also suppressed CaCl_2_-stimulated aorta contractions and led to the rightward shift of the response curves of a depolarized Ca^2+^-free medium, suggesting that the extract repressed extracellular Ca^2+^ influx and caused vasoconstriction. These outcomes strongly support the traditional use of *A. carambola* leaves in hypertension.

Orally administered ethanol extract of *A. carambola* roots (EEACR, at 150, 300, and 600 mg/kg) has been shown to lower blood pressure in healthy rats at 300 and 600 mg/kg but have no noticeable effect on the heart rate (Tang et al., 2017). When Huang et al. (2017) explored the effect of flavonoids from *A. carambola* fruit (FACF) on healthy rats and rats with NG-nitro-L-arginine-methyl ester (L-NAME)-induced high blood pressure, they established that FACF (at daily doses of 300, 600, and 1,200 mg/kg, i.g. for 5 weeks) significantly lessened systolic blood pressure, diastolic blood pressure, and mean blood pressure in healthy rats. Moreover, the blood pressure of rats with hypertension was also drastically lowered by 600 and 1,200 mg/kg FAC treatments, suggesting that FACF contains an active ingredient that decreases blood pressure (Table 2; Huang et al., 2017).

### Neuroprotective Activity

Alzheimer's disease (AD), characterized by the progressive deterioration of learning, memory, and cognition, is the most common, irreversible, and progressive neurodegenerative disease (Robins Wahlin and Byrne, 2011; Levenson et al., 2014). DMDD’s neuroprotective effect against memory deficits and neuron apoptosis in APP/PS1 transgenic AD mice has been reported (Wei et al., 2018). That research showed that mice receiving DMDD (at daily doses of 12.5, 25, and 50 mg/kg for 21 days) orally displayed significantly improved memory and spatial learning and inhibited neuron loss and apoptosis in APP/PS1 hippocampal tissues. *In vitro*, DMDD (at concentrations of 5, 10, and 20 μmol/L) radically suppressed Aβ1-42-stimulated apoptosis by upregulating Bcl-2 expression and downregulating Bax expression, increasing the mitochondria membrane potential (MMP) and activating PC-12 cell caspase-3 and caspase-9. Mice pretreated with DMDD also had drastically increased PC-12 cell Bcl-2/Bax ratio *in vitro* and *in vivo* (Wei et al., 2018). These findings indicated that DMDD has beneficial properties against the deficit of learning and memory in APP/PS1 transgenic AD mice through improving neuron apoptosis and suppressing Bax/Bcl-2-mediated loss of MMP (Table 2).

Recently, Lu et al. (2020) explored the neuroprotective activity of DMDD against Aβ1-42-evoked SH-SY5Y cell apoptosis and the underlying mechanism of DMDD’s protective function. Treatment with DMDD (at concentrations of 5, 10, and 20 μmol/L) significantly increased cell viability and inhibited Aβ1-42-induced SH-SY5Y cell apoptosis. Mechanistically, DMDD markedly downregulated Bax, caspase-3, caspase-8, and caspase-9 expressions, upregulated Bcl-2 levels, suppressed the release of cytochrome c, and the loss of MMP, and elevated the Bcl-2/Bax ratio (Lu et al., 2020). Overall, DMDD displayed excellent neuroprotective properties and is a promising ingredient for developing a neuroprotective drug for Alzheimer’s disease.

### Reducing Ultraviolet B-Induced Skin Damage

Ultraviolet B (UVB) is a major causation factor of cell injury and skin cancer. It causes DNA damage, promotes apoptosis, and produces ROS. In Ronpirin et al. (2016) investigation of the protective effect of *A. carambola* on HaCaT keratinocytes caused by UVB, the ethanol and aqueous fractions of *A. carambola* (EFAC and AFAC, at a concentration of 250 µg/ml) significantly decreased cell apoptosis. Both fractions also ominously lowered caspase 3 protein expression. Additionally, EFAC (at concentrations of 100 and 250 µg/ml) and AFAC (at concentrations of 50, 100, and 250 µg/ml) drastically reduced the percentage of cyclobutane pyrimidine dimers (CPD), resulting in DNA repair (Ronpirin et al., 2016). These reports suggest that *A. carambola* extracts could be developed cosmetically to protect against UVB-induced skin damage (Table 2).

### Antimicrobial Activity

The extract of the stem bark, leaves, and fruits of *A. carambola* supposedly display antibacterial and antifungal activity. Previous investigations demonstrated that the extracts of *A. carambola* fruits could suppress the growth of *Staphylococcus aureus* and *Klebsiella* spp., with the MBC of 15.62 and 125 mg/ml, respectively (Chang et al., 2000). Moreover, two compounds p-anisaldehyde and β-sitosterol (400 μg/disk) isolated from the bark of *A. carambola* strongly inhibited the growth of *Escherichia coli* with a zone of inhibition of 15 mm and had moderate inhibitory activity against fungi (Mia et al., 2007). Afterward, Wakte and Patil (2011) screened the antimicrobial activity of fruit extracts of star fruit at various stages against two Gram-positive bacteria (*S. aureus* ATCC 6538P and *B. cereus* ATCC 11778) and three Gram-negative bacteria (*E. coli* ATCC 25922, *P. aeruginosa* ATCC 19429, and *S. typhimurium* ATCC 23564), the results found that all the extracts show different degrees of activity against Gram-positive and Gram-negative bacteria, and the methanol and acetone extracts were considerably more effective than other solvent extracts in inhibiting the Gram-positive micro-organisms better than Gram negative microorganisms. In addition, the ethanolic extracts of *A. carambola* leaf at doses of 250 and 500 µg/disc were found to moderately suppress the growth of *Shigella dysenteriae*, *Streptococcus pyogenes*, *Staphylococcus saprophyticus*, *Streptococcus agalactiae*, and *Pseudomonas* spp. (Hossain et al., 2017).

Recently, Silva et al. (2021) found that the extracts of stem bark, leaves, and fruits from *A. carambola* perceptibly suppressed the growth of multi-resistant pathogenic bacteria and fungi with a minimal inhibitory concentration (MIC) of 100 μg/ml. Crude extracts of the leaf exhibited a broad spectrum of action against Gram-positive and Gram-negative bacteria, such as *S. aureus* 10 MRSA, *S. aureus* ATCC 29213 MRSA, *S. aureus* 12 MRSA, *S. aureus* 6 MRSA, *K. pneumoniae* 8 ESBL, *E. faecalis* ATCC 29212, and A*. baumannii* 2 MBL (Silva et al., 2021). Overall, the findings of these studies provide a research direction that points to *A. carambola*’s prospective therapeutic efficacy against bacterial and fungal infections.

## Toxicity Assessment

Literature material on the assessment of the toxicity and safety of *A. carambola* is limited, although this plant is commonly utilized in TCM to treat numerous ailments. In recent years, several studies have found that the excessive consumption of *A. carambola* results in toxic effects. Aranguren et al. (2017) literature review revealed that 123 patients from eight nations developed acute renal injury after consuming this fruit. Forty-seven of the cases were registered in Brazil, the highest reported incidence, followed by Taiwan (36), Bangladesh (20), China and France (8 each), Sri Lanka (2), and Thailand and Colombia (1 each); 28 of the patients died. In the most recent investigation, Yasawardene et al. (2021) analyses of 10 case series and 28 case reports in humans (total number of individuals = 136) and eight animal studies for *A. carambola* nephrotoxicity and neurotoxicity, 94 (69.1%) patients had prior renal impairment, with renal histology revealing acute oxalate nephropathy with tubulointerstitial nephritis or tubular necrosis. Also, neurotoxicity performances ranged from hiccups to status epilepticus. The excessive uptake of *A. carambola* could lead to acute kidney injury, particularly in an empty stomach, or dehydration state, and the use of *A. carambola* as therapy for an elderly patient is not recommended on an empty stomach (Wijayaratne et al., 2018). In addition, *A. carambola* has threatened people's health despite the relatively low frequency of star fruit intake. Further clinical studies on the toxic ingredients, metabolites, and intake dose of star fruit toxicity must be carried out to provide clear indications of intake and prevent toxicity and mortality.

The results of animal experiments showed that the high dose of the different *A. carambola* extracts showed different degrees of toxicity or adverse reactions. Chen et al. (2001) found that an overdose intake of the fruit could result in nephrotoxicity and neurotoxicity in some individuals (Chen et al., 2001). Supposedly, the fruit of *A. carambola* could cause fatality in patients suffering from uremia, with oxalate identified as being a significant element in the toxicity of the fruit (Dembitsky et al., 2011). Furthermore, an acute toxicity study conducted in female albino rats revealed that oral administration of *A. carambola* juice extract (ACJE) at different doses (250, 500, 1,000, 2000, 4,000, and 5,000 mg/kg) was safe and did not lead to any poisonous reactions after 48 h of treatment, despite the dosage reaching up to 5,000 mg/kg. In the subacute evaluations, oral ACJE (at dosages of 200, 400, and 600 mg/kg for 28 consecutive days) treatment elicited no significant difference in total protein, albumin, hematological parameters, and globulin values between treatment-receiving and control groups. However, serum AST, ALT, and ALP levels and urea, creatinine, and MDA levels in the treat-receiving group dose-dependently increased significantly more than in the control group. Moreover, when compared with the control group, the histomorphology of the livers and kidneys of the rats treated with ACJE displayed lesions of degeneration and necrosis (Aba and Amadi, 2019). Recent investigations systematically revealed that the nephrotoxic effect of *A. carambola* stems primarily from oxalate deposition in renal tubules, causing interstitial nephritis and acute tubular necrosis (Yasawardene et al., 2020). These results indicate that excessive and long-term consumption of ACJE could be nephrotoxic and hepatotoxic. Therefore, it is very important to study the intake dose as well as effective and safe dose in the future.

Phytochemical studies have shown that *A. carambola* contains two poisonous substances, caramboxin, and oxalic acid. Caramboxin is a non-proteinogenic amino acid that stimulates the glutamate receptors in neurons. The chemical structure of caramboxin is similar to the amino acid phenylalanine, it is metabolized and excreted through the kidneys (Yasawardene et al., 2020). Caramboxin can effectively stimulate the central nervous system (CNS), resulting in symptoms of CNS disorder, including mental confusion, seizures, and status epilepticus (Yasawardene et al., 2020). It can cause belch, vomiting, confusion, consciousness disorders, and shock, etc. If normal people eat this fruit, caramboxin can be safely discharged, so normal people will not be damaged by the toxin when it is ingested. However, the patients with renal insufficiency, especially in patients undergoing peritoneal dialysis or hemodialysis can’t eat it. Huynh and Nguyen (2017) findings showed that the alcohol fermentation technologies efficaciously reduced oxalate contents in *A. carambola*, preventing the risk of kidney stone formation. However, the works on the dose of caramboxin and the bioavailability of oxalate and caramboxin after ingestion are still insufficient, and more comprehensive, systematic, and authentic studies are required to assess the dose of caramboxin of star fruit juice and its pharmacokinetic parameters in healthy individuals.

## Future Outlooks

This review summarized the botany, ethnopharmacology, phytochemistry, pharmacology, and toxicity of *A. carambola* to explore this valuable fruit. The succulence and sweet taste of starfruit are of interest to the food industry. The pharmaceutical and health industries have been increasingly interested in *A. carambola* due to the nutritional properties as well as various health and pharmacological actions of this fruit. Phytochemical investigations have shown that **132** compounds have been mainly reported from *A. carambola*. The flavonoids represented by compounds **47** and **48** and benzoquinones represented by DMDD have been considered as the biologically active components with extensive biological properties, including anti-hyperglycemic, anti-hyperlipidemic, anti-obesity, anti-inflammatory, hepatoprotective, anti-tumor, cardioprotective, and neuroprotective activities. Numerous studies have revealed that *A. carambola* might be a promising candidate to treat diabetes mellitus and DN-related diseases. Furthermore, *A. carambola* can be employed in the prevention and treatment of ailments related to aging and oxidative stress. In summary, *A. carambola*, as a food and medicinal resource, has a good health care function and important edible and medicinal value, and thus has good prospects for utilization.

There are nevertheless still many problems in research that aims to bridge the gap between health and the bioactive substances of *A. carambola*, which need to be studied further: firstly, although there are a large number of *in vitro* and *in vivo* studies concentrated on the crude extracts of *A. carambola* using exorbitant doses, to the best of our knowledge, the active substances from these crude extracts with bioactivities are still unknown. Thus, research aiming to clarify the biological activities and the mechanism of active components found in *A. carambola* should be clearly conducted in further depth, as they will certainly collaborate to the establishment of safe dosages and accelerate the steps for the discovery of new molecules of biological interest. Secondly, the bioactive compounds and crude extracts of *A. carambola* have been increasingly and successfully utilized in diabetic disease prevention and health promotion in the last few decades. The documentary evidence points to a high number of bioactive compounds and crude extracts in star fruit that might be used as functional food sources against various illnesses including diabetes, cancer, cardiovascular disease, and other oxidative stresses or aging-induced chronic diseases. There is a need for further research to explore and utilize the processed products of this fruit, especially those phenolic extracts and dietary fibers in *A. carambola* for functional food formulation. Thirdly, although toxicity studies of *A. carambola* have been reported, the information related to its toxicity mechanism, especially nephrotoxicity and neurotoxicity, is still lacking. It is reported that *A. carambola* induced oxalate nephropathy remains an under-recognized cause for both acute and chronic kidney disease. Therefore, more public awareness about oxalate poisoning on uremic patients should be promoted. This will help to avoid adverse reactions to star-fruits in high uremic patients. The public must be well educated on the benefits as well as the hazardous effects of star fruits. Furthermore, toxicological studies are crucial for understanding the safety of herbal drugs. Therefore, to ensure the full use of its medicinal resources, further acute toxicity, and subacute toxicity, as well as the safety assessment studies of *A. carambola*, both *in vitro* and *in vivo*, should be carried out. Fourthly, continued efforts will be needed to clarify the pathways of pharmacokinetics including absorption, distribution, metabolism, and excretion, and to assess the long-term chronic toxicity and acute toxicity as well as the metabolites of phytochemicals formed *in vivo* of the bioactive compounds, especially for DMDD before proceeding to the development of pharmaceutical formulation. The pharmacokinetics parameters also contribute to facilitate the rational optimization of natural active substances, and increase therapeutic effects and reduce toxicities. Overall, in-depth research on clinical studies to justify their reported therapeutic potential, clinical efficacy, and safety of *A. carambola* is imminent.

Taken together, significant interest has been generated over *A. carambola* owing to the numerous beneficial effects. *A. carambola* appears to have great developing potential in the fields of functional food and modern medicine. Therefore, the opportunities and challenges co-exist. Meanwhile, it is an effective approach for TCM to get into the international market and an ideal choice for modern health care in developed countries for ongoing human health and thereby building a healthy community with a shared future for mankind. We believe that *A. carambola* will have an extensive international market and a broad prospect in terms of its applications in both medicine and functional food.

## Glossary

ACP: acid phosphatase
AI: atherogenic index
AIP: atherogenic index of plasma
AKP: alkaline phosphatase
ALP: alkaline phosphatase
ALT: alanine aminotransferase
AMPK α: AMP-activated protein kinase α
Ang II: angiotensin II
AST: aspartate aminotransferase
BAMBI: BMP and activin membrane bound inhibitor
Bax: bcl-associated X protein
BUN: blood urea nitrogen
cAMP: cyclic adenosine monophosphate
CAT: catalase
C/EBPα: CCAAT-enhancer binding protein α
Col III: collagen type III
Col IV: collagen type IV
CTGF: connective tissue growth factor
CVDs: cardiovascular diseases
CVF: collagen volume fraction
CysC: cystatin C
DENA: diethylnitrosamine
DMDD: 2-dodecyl-6-methoxycyclohexa-2,5-diene- 1,4-dione
DPPH: 2,2-diphenyl-1-picrylhydrazyl radical scavenging activity
eNOS: endothelial NO synthase
ECE: endothelin-converting enzyme
ET-1: endothelin 1
FAS: fatty acid synthase
FBG: fasting blood glucose
FFA: free fatty acids
FINS: fasting insulin
FRAP: ferric reducing antioxidant power
G-6-Pase: glucose-6-phosphatase
GC-MS: gas chromatography mass spectrometry
GHb: glucosylated hemoglobin
GSH: reduced glutathione
GSH-Px: glutathione peroxidase
HA: hyaluronic acid
HDL-C: high-density lipoprotein cholesterol
Hyp: hydroxyproline
iNOS: inducible NO synthase
IL-6: interleukin-6
ISI: insulin sensitivity index
LDL-C: low-density lipoprotein cholesterol
LN: laminin
LPO: lipid peroxidation
MDA: malondialdehyde
MPO: myeloperoxidase
Myd88: myeloid differentiation factor 88
NEFA: non-esterified fatty acid
NF-κB: nuclear factor-κB
p-AMPK α: phospho-AMPK α
PPAR-α/γ: peroxisome proliferator activated receptors-α/γ
Scr: serum creatinine
SCD1: stearoyl-CoA desaturase 1
SDH: sorbitol dehydrogenase
SGOT: serum glutamate oxaloacetate transaminase
SGPT: serum pyruvate transaminase
SOD: superoxide dismutase
SREBP-1c: sterol regulatory element binding protein-1c
STZ: streptozotocin
TAA: total ascorbic acid
TC: total cholesterol
TEAC: trolox equivalent antioxidant capacity
TG: triglycerides
TGF-β1: transforming growth factor beta 1
TL: total lipids
TLR4: Toll-like receptors-4
tNOS: total NO synthase
TNF-α: tumor necrosis factor-α
TQ: triglyceride
VLDL-C: very low-density lipoprotein cholesterol
